# Brain arteriovenous malformation in hereditary hemorrhagic telangiectasia: Recent advances in cellular and molecular mechanisms

**DOI:** 10.3389/fnhum.2022.1006115

**Published:** 2022-11-24

**Authors:** Elise Drapé, Typhaine Anquetil, Bruno Larrivée, Alexandre Dubrac

**Affiliations:** ^1^Centre de Recherche, CHU St. Justine, Montréal, QC, Canada; ^2^Département de Pharmacologie et de Physiologie, Université de Montréal, Montréal, QC, Canada; ^3^Département De Pathologie et Biologie Cellulaire, Université de Montréal, Montréal, QC, Canada; ^4^Département d’Ophtalmologie, Université de Montréal, Montréal, QC, Canada; ^5^Centre De Recherche, Hôpital Maisonneuve-Rosemont, Montréal, QC, Canada

**Keywords:** HHT, AVM, BMP, ALK1, ENG, SMAD4, endothelial cells

## Abstract

Hereditary hemorrhagic telangiectasia (HHT) is a genetic disorder characterized by vessel dilatation, such as telangiectasia in skin and mucosa and arteriovenous malformations (AVM) in internal organs such as the gastrointestinal tract, lungs, and brain. AVMs are fragile and tortuous vascular anomalies that directly connect arteries and veins, bypassing healthy capillaries. Mutations in transforming growth factor β (TGFβ) signaling pathway components, such as *ENG* (ENDOGLIN), *ACVRL1* (ALK1), and *SMAD4* (SMAD4) genes, account for most of HHT cases. 10–20% of HHT patients develop brain AVMs (bAVMs), which can lead to vessel wall rupture and intracranial hemorrhages. Though the main mutations are known, mechanisms leading to AVM formation are unclear, partially due to lack of animal models. Recent mouse models allowed significant advances in our understanding of AVMs. Endothelial-specific deletion of either *Acvrl1*, *Eng* or *Smad4* is sufficient to induce AVMs, identifying endothelial cells (ECs) as primary targets of BMP signaling to promote vascular integrity. Loss of ALK1/ENG/SMAD4 signaling is associated with NOTCH signaling defects and abnormal arteriovenous EC differentiation. Moreover, cumulative evidence suggests that AVMs originate from venous ECs with defective flow-migration coupling and excessive proliferation. Mutant ECs show an increase of PI3K/AKT signaling and inhibitors of this signaling pathway rescue AVMs in HHT mouse models, revealing new therapeutic avenues. In this review, we will summarize recent advances and current knowledge of mechanisms controlling the pathogenesis of bAVMs, and discuss unresolved questions.

## Introduction

Hereditary hemorrhagic telangiectasia (HHT), or Rendu-Osler disease, is an autosomal-dominant inherited syndrome with a prevalence of around 1:5,000–8,000 people (Bideau et al., 1989; Kjeldsen et al., 1999), characterized by vascular anomalies. The major lesions found are telangiectasia, widened small vessels located near the surface of the skin or mucous membranes such as lips, tongue, nasal, buccal, and gastrointestinal mucosa. These lesions are fragile and prone to bleeding mostly in the nasal mucosa and gastrointestinal tract (Shovlin, 2010). Most HHT patients also develop pulmonary, hepatic, spinal or brain arteriovenous malformations (AVMs), which are abnormal connections between arteries and veins. While 33% of patients develop pulmonary AVMs, 10–20% will develop brain AVMs (bAVMs) (Haitjema et al., 1995). Although bAVMs are less frequent, their consequences can be detrimental for the patient. Indeed, in addition to intracranial hemorrhages, bAVMs can lead to blood-brain-barrier (BBB) defects, promoting neuronal dysfunction and seizure such as epilepsy (Mohr et al., 2013; Rohn et al., 2014). However, bAVM pathogenesis is not well understood.

**FIGURE 1:**
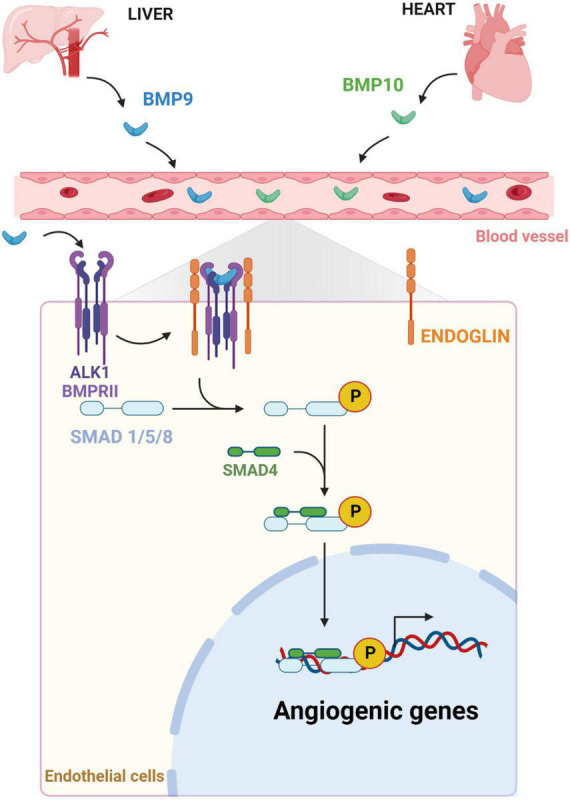
Endothelial BMP/ALK1 signaling pathway. Soluble BMP9 and BMP10, secreted, respectively, by the liver and the heart in the bloodstream, bind their receptor ALK1, a SER/THR kinase, and its coreceptors BMPRII and ENDOGLIN at the surface of ECs. The activation of the BMP receptor complex will trigger the downstream SMAD signaling. Phosphorylated SMAD1/5/8 will recruit SMAD4. This SMAD complex accumulates in nucleus to regulate transcription of target genes, including genes involved in angiogenic processes.

It is now well known that ALK1 signaling, one of the canonical pathways of the TGFβ superfamily, plays a critical role in vascular morphogenesis (Roman and Hinck, 2017). BMP9 and BMP10 are cytokines produced by the liver and the heart, respectively (Figure 1; Neuhaus et al., 1999; Bidart et al., 2012). BMP9 expression has also been reported in the lung and the brain septum, although at a significantly lower level than in the liver (López-Coviella et al., 2000; Bidart et al., 2012). Cardiac BMP10 expression is associated with the development of the trabeculated myocardium during embryonic development and then becomes mainly restricted to the right atria in post-natal life (Neuhaus et al., 1999; Somi et al., 2004). These cytokines can have an autocrine and paracrine action but can also enter blood circulation to act on endothelial cells (David et al., 2008; Chen H. et al., 2013). Indeed, BMP9/10 bind with high affinity to the TGFβ receptor 1 ALK1, a serine/threonine kinase receptor, and its coreceptor, ENDOGLIN (Figure 1), which is expressed predominantly on ECs (David et al., 2006; Alt et al., 2012; Townson et al., 2012). BMP9/10 ligands bind to ALK1 which will heterodimerize with BMP receptor II (BMPRII) to transduce downstream signaling. The ENDOGLIN receptor interacts with the receptor complex to promote the phosphorylation of the transcription factors SMAD1/5/8 (Chen and Massagué, 1999; Oh et al., 2000), leading to their association with the common regulator SMAD4 (Nakao et al., 1997). Then, the SMAD complex translocates and accumulates into the nucleus (Figure 1) in order to regulate the expression of specific target genes such as DNA-binding protein inhibitor 1 (*ID1*) and *ID3* (Valdimarsdottir et al., 2002). For example, the BMP9/ALK1 signaling pathway regulates angiogenesis by modulating the expression of genes involved in vascular quiescence, cell junction, response to shear stress, and the recruitment of mural cells.

HHT patients can be subdivided into five groups. Mutations in the *ENG* gene (encoding for ENDOGLIN) are responsible for HHT1 (McAllister et al., 1994), while mutation of *ACVRL1* (encoding for ALK1) leads to HHT2 (Johnson et al., 1996). HHT1 and HHT2 represent around 90% of HHT cases (Prigoda et al., 2006). Moreover, mutations of *SMAD4* represent 2% of a type of HHT called juvenile polyposis (JP)-HHT (Howe et al., 1998; Gallione et al., 2004). Finally, loci on chromosomes 5 and 7, unlinked to *ENG*, *ACVRL*1, or *SMAD4* mutations, have been associated with subsets of HHT patients and have been referred to as HHT3 and HHT4, respectively (Cole et al., 2005; Bayrak-Toydemir et al., 2006), while HHT features (HHT5) have also been observed in individuals with heterozygous mutations of growth differentiation factor 2 (GDF2), which encodes BMP9 (Wooderchak-Donahue et al., 2013). It should be noted that the vascular phenotype varies depending on the mutated HHT genes but also on the individual. Indeed, mutations in the same gene can trigger different vascular anomalies from one patient to another, suggesting context-dependent pathogenicity of vascular malformations (Kjeldsen et al., 2005; Bayrak-Toydemir et al., 2006).

This review will focus on HHT bAVMs and summarize recent advances in organotypic pathogenicity, animal models, cellular and molecular mechanisms, and therapeutics.

## Cerebral arteriovenous malformation anatomy

### Brain vasculature

The blood supply of the human brain relies on only two pairs of large arteries: the internal carotids, which supply the blood to the cerebrum, and the vertebral arteries, which join distally to form the basilar artery, and finally vascularize the cerebellum and the brain stem. Proximally, the basilar artery and the internal carotid arteries join to form a ring at the brain’s base known as the circle of Willis.

The circle of Willis gives rise to three pairs of main arteries: the anterior, middle, and posterior cerebral arteries, which divide progressively into smaller arteries and pial arterioles within the brain’s subarachnoid space meninges. Then, penetrating arterioles dive into the cortex and divide into precapillary arterioles and capillaries, the most predominant brain vessels. Similarly, the pial venular network from the brain surface is connected to ascending venules diving into the parenchyma, followed by postcapillary venules, which branch to capillaries.

Even if human brain vascularization is well described, its development is based on studies done in animal models, such as rodents. Comprehensive studies on the mouse brain showed that vascularization starts at embryonic day (E) 7.5–8.5 by vasculogenesis at the ventral region of the neural tube, followed by the invasion of capillary sprouts into the neuroepithelium at E9.5 (Hogan et al., 2004). Branching and arborization of capillary sprouts from the pial surface subsequently lead to the formation of the perineural vascular plexus (PNVP) at the brain’s surface (Tata et al., 2015; Biswas et al., 2020). Then, the first sprouts of the PNVP invade the ventrolateral forebrain following an angiogenic gradient of Vascular Endothelial Growth Factor (VEGF) induced by the hypoxic tissue at postnatal day (P) 9.5 (Bautch and James, 2009; Tata et al., 2015; Peguera et al., 2021). The vascularization will then continue to invade the tissue in a ventrolateral to dorsomedial fashion by angiogenesis, the growth of pre-existing blood vessels. The cerebral vascular network is still expanding and remodeling for a few weeks after birth (Biswas et al., 2020). While canonical pro-angiogenic signaling pathways, such as VEGF, WNT, and TGFß, are essential, mechanisms controlling this postnatal angiogenesis wave remain unclear (Paredes et al., 2018). Neo-angiogenic sprouts and EC proliferation are observed until P14. Then, from P14 to P25, a progressive decrease in the angiogenic rate will favor the stabilization of the vasculature (Biswas et al., 2020; Coelho-Santos and Shih, 2020). Moreover, the capillary density varies depending on the brain region. Indeed, the angiogenesis rate is far higher in gray matter, enriched in neuronal cell bodies, than in white matter to supply the energy needed (Coelho-Santos and Shih, 2020).

The ECs lining vessels have developed a unique and highly selective BBB to maintain brain homeostasis and neural function (Daneman and Prat, 2015). This organotypic feature of the CNS capillaries depends on the interaction and communication between the neurovascular unit (NVU) cells, which includes ECs, pericytes (PCs), astrocyte endfeet as well as neurons (Siegenthaler et al., 2013; Kaplan et al., 2020). In the mouse, brain angiogenesis begins at E9.5-E11.5, and the initiation of BBB starts soon after (Biswas et al., 2020). ECs acquire both paracellular and transcellular barriers to prevent the movement of small molecules between ECs (Daneman and Prat, 2015; Biswas et al., 2020). The endothelial BBB is characterized by abundant tight junctions (paracellular barrier) and a low rate of endocytosis (transcellular barrier) to control passive exchanges and caveolae-dependent receptor-mediated transcytosis. Therefore, the BBB allows a high control of the nutrient exchange, low permeability, and immune privileges (Siegenthaler et al., 2013; Daneman and Prat, 2015; Haddad-Tóvolli et al., 2017).

### Brain AVMs anatomy

A healthy blood vascular network has capillary plexuses between arteries, a high flow region, and veins, a low flow region. Capillaries are essential for the delivery of oxygen and nutrients. Therefore, neurovascular coupling, the blood flow regulation by neurons, is essential for neuronal function (Kaplan et al., 2020). The main characteristic of AVM is a direct connection between veins and arteries, bypassing the capillary bed. The loss of capillaries in bAVMs leads to cerebral hemodynamic changes with the increased blood flow in the arteries and veins. Thus, bAVMs are considered high-flow vascular malformations. bAVMs are also characterized by one or several feeding arteries and one draining vein. Moreover, bAVMs are associated with ECs proliferation and mural cell coverage defects.

There are different types of bAVMs defined by specific features (Matsubara et al., 2000): arteriovenous fistulas (AVF), nidal AVMs, and capillary vascular malformations (CVM). AVFs correspond to direct fistula connections between one artery and one vein, whereas nidal AVMs and CVMs are characterized by the presence of a nidus, defined as tangles of abnormal blood vessels. Nidal AVMs have a nidus between 1 and 3 cm with multiple feeding arteries and veins, whereas the nidus of CVM is less than 1 cm and has one feeding artery and vein (Matsubara et al., 2000; Griauzde et al., 2020). Patients with AVF and nidal AVM have more chance of rupture risk and may present symptoms such as seizure, hemorrhage, and headache, whereas most patients with CVM are asymptomatic (Matsubara et al., 2000; Brinjikji et al., 2017). The different types of bAVMs can also be discerned by the age of the patients. Indeed, AVFs are observed in young children, whereas nidus AVMs and CVMs develop later in life (Matsubara et al., 2000; Krings et al., 2005).

Even if the different AVMs have different characteristics, no differences in the localization in the brain have been reported (Krings et al., 2005). AVMs are located mostly in superficial regions of the cerebral cortex, with a majority in the supratentorial region compared to the infratentorial region. However, very few AVMs can be found in deeper areas such as the basal ganglia or brain stem (Matsubara et al., 2000; Saleh et al., 2013; Komiyama et al., 2015; Krings et al., 2015).

### Consequences and symptoms of brain AVMs

Most bAVMs are asymptomatic, and the diagnosis is often made by brain imaging after hospitalization or examination for another medical reason. However, some patients may develop symptoms such as headaches, seizures, and hemorrhages (Brown et al., 1996). In addition, it seems that there is a correlation between the hemodynamic changes, the patients’ age, and the AVM’s cerebral location (Gross and Du, 2013; Abecassis et al., 2014). The exact causes of seizures are not yet well understood. However, frontal or temporal lobe locations of AVMs are associated with a higher risk of seizure compared to a deep location (Hoh et al., 2002). In addition, it also appears that hemodynamic changes may influence the occurrence of seizures (Fierstra et al., 2011).

The risks of hemorrhage may be increased by the position of the AVM in the brain, with a deep location having a higher risk of rupture (Yamada et al., 2007). However, the bAVMs found in HHT patients predominantly have a cortical area and therefore have a relatively low risk of rupture (around 2% per year) (Willemse et al., 2000). Moreover, it has also been documented that the size of the AVM and its association with aneurysms may also influence the risk of hemorrhage (Gross and Du, 2013). The high blood pressure generated by bAVM may also increase the risk of rupture (Langer et al., 1998). Hemorrhage after bAVM rupture leads to mortality or morbidity development (Fleetwood and Steinberg, 2002). Indeed, it has been shown that patients with hemorrhagic bAVMs have a higher risk of subsequent epileptic seizure (Josephson et al., 2011).

However, mechanisms that lead to the development of those symptoms are not yet well understood, mainly because only a few patients develop cerebral symptoms, the distribution of these symptoms are heterogeneous among patients, and the lack of experimental models.

## Animal models of hereditary hemorrhagic telangiectasia

### Mouse models

It is crucial to develop animal models that recapitulate HHT pathophysiology to improve our knowledge of AVMs’ pathogenesis and reveal new therapeutic avenues. Several studies have used new sophisticated models to decipher AVM mechanisms in the last two decades, as summarized below (Tual-Chalot et al., 2015).

Three transgenic mouse models with global knockout for *Eng* were first developed in order to recapitulate HHT1 (Bourdeau et al., 1999; Li et al., 1999; Arthur et al., 2000). These mice show marked defects in yolk sac angiogenesis and cardiac development, leading to lethality at E10.5. Similarly, global knockout mice for *Acvrl1* have been developed to study HHT2 (Oh et al., 2000; Urness et al., 2000; Srinivasan et al., 2003). These mice also die around E10.5 with pronounced angiogenic defects. Indeed, mutant embryos exhibit hyperdilated vessels and the formation of arteriovenous shunts. The embryonic lethality of these mutant mice highlights the importance of the BMP/ALK1/ENG signaling pathway in vascular development and morphogenesis but makes it challenging to study the mechanisms underlying these angiogenic defects. Thus, the following studies were carried out on heterozygous mice. In addition, to be viable, these models are closer to HHT physiopathology, as HHT patients also carry heterozygous mutations (Srinivasan et al., 2003; Torsney et al., 2003). Moreover, heterozygous mutant mice display some features found in patients, such as mucocutaneous vascular lesions in internal organs, such as lungs, liver, spleen, and intestines. However, these mice develop very few cerebral AVMs at a lower frequency than that found in patients.

The creation of inducible and tissue-specific mutant mice was crucial for the investigation of the role of key genes in developmental and pathological processes. Reporter mice have shown that ALK1 and ENG are mainly expressed in ECs of blood vessels (Jonker and Arthur, 2002; Seki et al., 2003). In line with these findings, the endothelial-specific *Acvrl1* deletion is sufficient to trigger blood vessels dilation, hemorrhages and AVMs in the brain, lungs and intestine (Park et al., 2009). However, endothelial mutant mice showed improved survival until postnatal day 5 compared to global KO which induces embryonic lethality. New inducible and endothelial-specific models allowed studying AVMs in postnatal mice. Interestingly, an early postnatal endothelial-specific deletion of *Eng* or *Acvrl1* is sufficient to induce an HHT-like phenotype, including AVM (Table 1; Allinson et al., 2007; Park et al., 2008, 2009; Mahmoud et al., 2010). However, the deletion of *Eng* in perivascular smooth muscle cells does not promote arteriovenous shunt formation, in line with its expression profile (Garrido-Martin et al., 2014). Altogether, these findings revealed the essential role of ECs in the pathogenesis of HHT. These models also confirmed the hypothesis that a second hit is necessary to induce AVM in HHTs, also known as the three events hypothesis (Tual-Chalot et al., 2015). This refers to the fact that 3 stimuli are necessary for AVM formation: gene loss, protein loss, and then an angiogenic stimulus. While an early endothelial deletion of *Eng* and *Acvrl1* promotes AVMs formation in the angiogenic context of developing tissues, the deletion in adults is not sufficient unless there is an angiogenic or inflammatory stimulus. Indeed, local injection of VEGF or LPS following an endothelial *Acvrl1* or *Eng* deletion induces several vascular anomalies including bAVMs in adult mice (Walker et al., 2011; Choi et al., 2012, 2014; Han et al., 2014). In addition, AVMs also develop in the skin of adult *Acvrl1* mutant mice when angiogenesis is induced with a wound, proving the necessity of an angiogenic hit for AVM formation (Park et al., 2009).

**TABLE 1:** Summary of the major HHT mouse models.

Target genes	Deletion site	CRE-line	Brain/Retina phenotype	Other phenotype	References
*Eng*	All cell type	–	–	Homozygous deletion: Lethality at E.10 - E.10.5/Altered angiogenesis in the yolk sac/Defects in cardiac development	Bourdeau et al., 1999; Li et al., 1999; Arthur et al., 2000; Torsney et al., 2003
				Heterozygous deletion: Viable/Telangiectasia in the nose, mouth and ears/Hemorrhages/Subcutaneous AVMs	
*Acvrl1*	All cell type	–	–	Homozygous deletion: Lethality at E.10 - E.10.5/Angiogenesis defect/Vessel hyperdilatation/Arterio-venous shunt	[Bibr B121][Bibr B174]; Srinivasan et al., 2003
				Heterozygous deletion: Viable/Cutaneous, mucocutaneous and internal organ vascular defects (Vessel dilatation)	
*Eng*	Endothelial cell	*Cdh5-CRE^*ERT*2^*	Postnatal deletion: Dense capillary plexus increase ECs proliferation and AVMs formation in the retina.	Adult deletion: Angiogenesis defect in subdermal matrigel assay with vessel dilatation	Mahmoud et al., 2010
*Eng*	Endothelial cell	*Scl-CRE^*ERT*2^*	–	Adult deletion: AVMs formation after skin injury	Garrido-Martin et al., 2014
*Eng*	Venous and capillary endothelial cell	*Apj-CRE^*ERT*2^*	Postnatal deletion: AVMs formation in retina	–	Singh et al., 2020
*Acvrl1*	Endothelial cell	*Scl-CRE^*ERT*2^*	–	Adult deletion: AVMs formation after skin injury, in gastrointestinal tract and hemorrhages in caecum	Garrido-Martin et al., 2014
*Acvrl1*	Endothelial cell	*Cdh5-CRE^*ERT*2^*	Postnatal deletion: Hyperbranching, ECs proliferation and AVMs formation	Postnatal deletion: Lung hemorrhages	Tual-Chalot et al., 2014
				Adult deletion: Ceacal hemorrhages	
*Acvrl1*	Endothelial cell	*Pdgfb-CRE^*ERT*2^*	Adult deletion: bAVMs formation after VEGF stimulation	–	Chen W. et al., 2014
*Acvrl1*	Venous and capillary endothelial cell	*Mfsd2a-CRE^*ERT*2^*	Postnatal deletion: brain and retinal AVMs	Postnatal deletion: Gastrointestinal AVMs	Park H. et al., 2021
*Acvrl1*	Arterial endothelial cell	*Bmx-CRE^*ERT*2^*	Post-natal deletion: no AVMs in brain or retina	–	Park H. et al., 2021
*Acvrl1*	Tip cell	*Esm1-CRE^*ERT*2^*	Post-natal deletion: Vascular malformation in retina and brain but no AVM	Post-natal deletion: AVMs in intestinal villi and mesenteries	Park H. et al., 2021
*Smad4*	Brain endothelial cell	*SP-A-CRE*	Embryonic deletion: brain hemorrhages, BBB breakdown and ECs proliferation	–	Li et al., 2011
*Smad4*	Endothelial cell	*Cdh5-CRE^*ERT*2^*	Postnatal deletion: bAVMs and retinal AVMs associated with ECs proliferation and change in arterio-venous identity	Postnatal deletion: Gastrointestinal AVMs	Ola et al., 2018
*Smad4*	Venous endothelial cell	*Gm5127-CRE^*ERT*2^*	Postnatal deletion: AVMs formation in retina with ECs proliferation	–	Lee et al., 2021
Bmp9/10	Blood circulation (Blocking Ab)	–	Postnatal deletion: Hypervascularization and AVMs formation in the retina	–	Ruiz et al., 2016; Baeyens et al., 2016; Ola et al., 2016
*Eng*	Smooth muscle cell	*Myh11*-CRE^*ERT*2^	Adult deletion: No AVM formation	Adult deletion: No AVM formation	Garrido-Martin et al., 2014
*Acvrl1*	Smooth muscle cell	*Myh11*-CRE^*ERT*2^	Adult deletion: No AVM formation	Adult deletion: No AVM formation	Garrido-Martin et al., 2014
*Acvrl1*	Smooth muscle cell	*Tagln-CRE*	Embryonic deletion: AVM formation in the brain and microhemorrhages	–	Han et al., 2021

In patients, heterozygous mutations in *Smad4* lead to JP-HHT, but the mechanisms are still poorly understood. Similar to *Acvrl1* and *Eng* knockout mice, a global loss of *Smad4* results in lethality at the E10.5 (Lan et al., 2007). Constitutive endothelial *Smad4* deletion decreases blood vessel integrity and leads to cerebral bleeding, specifically during embryonic development (Li et al., 2011). In addition, postnatal loss of *Smad4* induces the formation of AVMs in the retina, brain, and gastrointestinal tract, phenocopying vascular phenotypes observed in endothelial *Acvrl1* and *Eng* mutant mice (Table 1; Ola et al., 2018).

Mutations in the GDF2 gene are responsible for less than 1% of HHTs (Wooderchak-Donahue et al., 2013; Hernandez et al., 2015). In mice, injection of antibodies blocking both BMP9 and BMP10 has been shown to phenocopy endothelial *Eng*, *Acvrl1*, and *Smad4* mutant mice to some degree, with AVM formation in the retina and the gastrointestinal tract, suggesting that it could be a model of HHT (Table 1; Baeyens et al., 2016; Ola et al., 2016; Ruiz et al., 2016, 2020). Moreover, while deletion of either *Gdf2* or *Bmp10* does not promote vessel dilatation and AVM formation (Ricard et al., 2012), postnatal double *Gdf2* and *Bmp10* deletion induces vascular anomalies, including AVMs in the gastrointestinal tract (Bouvard et al., 2021).

Recently, new genetic tools have been developed to delete genes in specific EC subtypes, such as capillaries, veins, arteries, and tip cells, to study the onset and progression of AVMs. Therefore, it has been shown that deletion of *Eng* (Singh et al., 2020), *Smad4* (Lee et al., 2021), and *Acvrl1* (Park H. et al., 2021) in venous ECs is sufficient to induce AVMs, unlike deletion in other EC subpopulations (Table 1). The underlying mechanisms will be discussed in later sections.

Despite the development of new mouse models, the study of bAVMs remains complicated due to their 3D structure and unpredicted location in the brain. Therefore, many studies have been performed on the retina which has a vascular network growing in 2D that can be imaged in wholemount. Moreover, retina and brain blood vessels share numerous features including the NVU and Blood Retina Barrier (BRB). The retina is a part of the central nervous system and is a well-established model for studying angiogenic processes such as endothelial sprouting, arterio-venous specification, and vascular remodeling (Adams and Alitalo, 2007; Fonseca et al., 2020). In mice, the vascularization of the retina starts at birth, and blood vessels grow from the optic nerve to the periphery until P7 to form a superficial vascular plexus. Then, blood vessels dive into the neuroretina to form the deep vascular plexus until P12, followed by the formation of the intermediate plexus until P15, and the vascular network is completed and fully mature at P21 (Gariano and Gardner, 2005). Compared to the brain, the superficial retinal plexus is a stereotypic vascular network that can be imaged by wholemount, allowing the study of complex vascular structures such as AVMs. Therefore, we and others have shown that endothelial deletion of *Eng*, *Acvrl1*, or *Smad4* induces AVMs in mouse retinas, which has greatly improved our understanding of the mechanisms involved in the pathogenesis (Mahmoud et al., 2010; Tual-Chalot et al., 2014; Baeyens et al., 2016; Ola et al., 2016, 2018; Crist et al., 2018).

All this work shows that endothelial deletion of *Eng*, *Acvrl1* and *Smad4* are appropriate models to study pathology. In the retina, in addition to the formation of AVMs, mutants show an increase in EC sprouting, proliferation, and vessel dilatation. Mural cells are also affected with a decrease in pericyte coverage on the one hand and arterialization of the veins on the other with the recruitment of vascular smooth muscle cells (vSMC). However, despite some degree of similarity in the vascular phenotype, these mutant mice also present distinct phenotypes. For example, 60% of *Acvrl1* mutants develop AVMs in the retina (Tual-Chalot et al., 2014), whereas this percentage is around 70 and 82% in *Eng* and *Smad4* mutants, respectively (Mahmoud et al., 2010; Crist et al., 2018). Moreover, both *Eng* and *Smad4* mutants exhibit a delay in vessel outgrowth but not *Acvrl1* mutants. *Acvrl1* mutants show a hyperbranching more pronounced than *Eng* and *Smad4* mutants, which is associated with a strong increase in the number of tip cells. However, it should be noted that the dose of tamoxifen used and the age of the mice at time of injection could also influence these differences. Further investigation is required to accurately compare vascular phenotypes of those models.

Overall, these new transgenic mouse models showed that the BMP/ALK1/ENG/SMAD4 signaling pathway is essential for both developmental and adult vascular morphogenesis. Indeed, in addition to AVMs, the postnatal endothelial deletion of *Eng*, *Acvrl1*, and *Smad4* are lethal, and survival varies depending on the gene and age.

### Zebrafish models

Zebrafish also represent an important model for studying bAVMs. First, because of the high degree of homology between zebrafish and vertebrates regarding vasculature and angiogenic mechanisms (Schuermann et al., 2014). Secondly, thanks to the transparency of the embryos it is easy to follow the vasculature and the development of vascular malformation using fluorescence imaging. Finally, the facility to perform genetic mutations makes the zebrafish an essential model for studying vascular development and disease. *acvrl1* mutation in zebrafish induces cranial vessel defects and enlargement, ultimately leading to the formation of AVMs with direct connections between the basilar and basal communicating arteries with the primordial midbrain and hindbrain veins (Roman et al., 2002; Corti et al., 2011). Moreover, *bmp10* mutants phenocopy *acvrl1* mutants, with vessel enlargement, increase of ECs number and formation of direct connection between arteries and the midbrain and hindbrain veins (Laux et al., 2013). Indeed, *bmp10* mutants exhibit blood vessel abnormalities in liver, heart dysmorphology, vascular defects correlating with increased cardiac output and embryonic lethal cranial AVMs indistinguishable from *acvrl1* mutants (Capasso et al., 2020), highlighting that in zebrafish, *bmp10* encodes the only required Alk1 ligand in the juvenile-to-adult period. By contrast, *bmp9* mutants survive to adulthood and do not develop cranial AVMs (Capasso et al., 2020), although they do display transient remodeling defects of the caudal venous plexus (Wooderchak-Donahue et al., 2013). Recently, Sugden et al. (2017) have shown that zebrafish mutated for *eng* at the embryonic stage survive, whereas they develop AVM-like vascular defects. The authors also showed that loss of *eng* in adults leads to dilation of cerebral vessels, and that only induction of an angiogenic hit through fin amputation lead to bAVM formation (Sugden et al., 2017). Finally, the contribution of Smad signaling in AVM formation was also highlighted in zebrafish by a study showing that *Smad9* morphants develop cranial AVMs with morphologic similarities to human bAVMs (Walcott et al., 2018).

## Hereditary hemorrhagic telangiectasia and BMP/ALK1 signaling pathway

AVMs develop in high blood flow regions and are often associated with vascular proliferation. The ECs lining blood vessels are exposed to blood flow which induces mechanical forces. The generated laminar shear stress (LSS), within the physiological range, triggers a number of flow responses in EC, including PI3K/AKT signaling pathway activation, alignment in the direction of flow (Tzima et al., 2003; Coon et al., 2015), migration against the blood flow (Parmar et al., 2006; Ouarné et al., 2021), inhibition of proliferation (Akimoto et al., 2000), and PCs recruitment (Van Gieson et al., 2003). Therefore, LSS is essential for blood vessel integrity, vascular remodeling, and morphogenesis. Recent studies have demonstrated an essential role for the BMP/ALK1/ENG signaling pathway in the LSS-induced PI3K/AKT activation and vascular quiescence (Laux et al., 2013; Ola et al., 2016, 2018; Jin et al., 2017). Indeed, PI3K/AKT signaling pathway is an important regulator of EC proliferation (Graupera and Potente, 2013), and it can be inhibited to promote vascular quiescence. Therefore, the understanding of the BMP/ALK1/ENG-LSS crosstalk could allow the identification of new therapeutic strategies.

### Cellular and molecular regulation of hereditary hemorrhagic telangiectasia arteriovenous malformations

#### HHT1: *ENG* mutation

Angiogenic ECs express high levels of *Eng* in development and disease, whereas its expression is attenuated in adult quiescent endothelium (Miller et al., 1999; Jonker and Arthur, 2002; Torsney et al., 2002). Moreover, *Eng* depletion inhibits TGFβ-induced proliferation arrest *in vitro* and *in vivo*, resulting in hyperproliferation and migration defects (Park et al., 2013).

In endothelial *Eng* mutant mice mimicking HHT1, 70% of retinas develop multiple AVMs and bleeding. These retinas also exhibit several other vascular anomalies, such as increased vascular branching, sprouting, vessel diameter, and endothelial proliferation (Mahmoud et al., 2010). Loss of *Eng* induces arterial and venous identity defects, and it can also be noted that AVMs are positive for venous markers such as EPHRIN B4 (*EphB4*) and APELIN receptor (*AplnR*). Blood flow was shown to promote the association of ALK1 and ENG at the EC membrane, thereby enhancing BMP signaling and vascular quiescence (Baeyens et al., 2016). A recent study showed that *Eng* mutant ECs fail to migrate against the flow and exhibit an increase in VEGF-induced PI3K/AKT activation (Jin et al., 2017). Inhibition of VEGFR2 or PI3K/AKT signaling decreased the number of AVMs in *Eng* mutant retinas. In addition, the authors also showed that *Eng* mutant ECs tend to proliferate mainly in arteries. One study in which *Eng* was specifically deleted only in capillaries and veins but not in arteries (Singh et al., 2020) showed that these mice still have AVMs in the retinas, suggesting that the loss of *Eng* in arteries is not involved in the development of AVMs. Similar observations have been made in zebrafish, where *eng* mutations lead to the formation of AVMs with an increase in vessel diameter (Sugden et al., 2017) caused by defects in flow response and cell polarization leading to vessel enlargement. The role of *Eng* in the flow response and the pathogenesis of AVMs, therefore, appears to be a conserved process. Altogether, these studies demonstrate that *Eng* is essential for EC specification, migration against the flow, and shear stress-induced quiescence to prevent AVMs formation.

#### HHT2: *ACVRL1* mutation

In the HHT2 mouse model, the endothelial *Acvrl1* deletion induces an increase in vascular density, endothelial sprouting as well as the formation of AVMs in the retina (Park et al., 2009; Larrivée et al., 2012; Ola et al., 2016). Similar to HHT1 models, endothelial *Acvrl1* deletion decreases PC recruitment, arterial marker expression such as JAGGED1, and AVMs are positive for the venous marker EPHRIN B4. Interestingly, retinal shunts develop near the optic nerve in high flow regions, while hyperbranching and hypersprouting develop mainly at the migration front, a low flow region (Bernabeu et al., 2014; Baeyens et al., 2016). These data suggest that, like the HHT1 models, LSS is important in the development of HHT2’s AVMs. Moreover, several studies in zebrafish have shown that *acvrl1* expression is induced by blood flow (Corti et al., 2011; Laux et al., 2013). *acvrl1* deletion also decreased the expression of genes involved in the flow-response, such as *Edn1* and *Cxcr4.* However, the expression of *Klf2*, a transcription factor essential for the endothelial flow-response (Parmar et al., 2006), is not altered. Although endothelial *Acvrl1* inhibition promotes arterial enlargement associated with an increase in the number of ECs, a recent study showed AVMs formation requires mutant capillary and venous and not arterial ECs (Park H. et al., 2021). While arterial-specific *Acvrl1* deletion did not induce obvious vascular phenotypes and lethality, deletion in venous ECs results in retinal AVMs and is lethal 5–6 days after gene deletion. Moreover, *Acvrl1* inhibition in endothelial tip cells, cells leading vascular sprouts and arteries formation (Xu et al., 2014; Pitulescu et al., 2017), failed to induce retinal AVMs (Park H. et al., 2021). However, these mutant mice develop capillary malformations in the retina, intestinal villi, and brain, and they die 10–11 days after tamoxifen injection, suggesting that they might develop AVMs in other tissues. Therefore, Park H. et al. (2021) proposed that HHT2’s AVMs originate from defective venous ECs. Indeed, mutant ECs present a polarization defect and migrate randomly instead of being directed against the blood flow. This phenomenon has also been observed in the zebrafish model (Rochon et al., 2016).

Skin biopsies of HHT2 patients showed an increase in the PI3K/AKT pathway suggesting an involvement of this pathway in AVM formation (Alsina-Sanchís et al., 2018). *In vitro*, BMP9 stimulation blocks VEGF-induced EC proliferation, and this effect is lost with the *ACVRL1* knockdown. Moreover, inhibition of *ACVRL1* increased VEGF-induced VEGFR2 activation and AKT phosphorylation (Ola et al., 2016; Alsina-Sanchís et al., 2018). While VEGFR2 is the main VEGF receptor that mediates VEGF-induced EC proliferation and angiogenesis, VEGFR1 cannot transduce VEGF signaling and acts as a decoy receptor. Therefore, VEGFR1 traps VEGF ligand, and VEGFR1 inhibition increases VEGF bioavailability and signaling. Interestingly, patients and mouse models of HHT2 show decreased VEGFR1 expression. Thalgott et al. (2018) have recently shown that blocking VEGFR1 induces retinal AVMs in a VEGFR2-dependent manner. In addition, VEGFR2 inhibition, using DC101 blocking antibody, can also rescue the formation of AVMs, as well as hypersprouting and hyperbranching, in *Acvrl1* mutant retinas highlighting that exacerbated VEGF signaling is associated with AVM formation in *Acvrl1* mutant models (Ola et al., 2016).

Pharmacological or genetic inhibition of the PI3K/AKT signaling pathway also rescues EC proliferation and AVMs in *Acvrl1* mutant mice (Ola et al., 2016; Alsina-Sanchís et al., 2018), demonstrating the contribution of this signaling pathway in the development of AVMs. PTEN is a phosphatase that negatively regulates the PI3K/AKT pathway by modulating the phosphatidylinositol 3,4,5-trisphosphate (PIP3) conversion into phosphatidylinositol 4,5-bisphosphate (PIP2) (Graupera and Potente, 2013). *In vitro* studies have shown that BMP9/ALK1 signaling increase PTEN activity in ECs, thereby decreasing PI3K/AKT signaling (Ola et al., 2016; Alsina-Sanchís et al., 2018). However, while the loss of endothelial PTEN in ECs increases EC proliferation and induces retinal vascular hyperplasia, it does not induce AVM formation (Serra et al., 2015).

LSS-induced EC polarity and migration are regulated by integrin binding to extracellular matrix proteins and signaling to CDC42 (cell division control protein 42 homolog) (Etienne-Manneville and Hall, 2001; Tzima et al., 2003). Integrins are transmembrane receptors that allow the adhesion of ECs to the extracellular matrix, which is an important process for angiogenesis (Serini et al., 2008). Some integrins interact with VEGFR2 at the EC membrane regulating its activity and the activation of the PI3K/AKT and YAP/TAZ signaling pathways. A recent study showed that *Acvrl1* deletion increased the expression of integrin β1, α5, and αv in AVMs (Park H. et al., 2021). They also found an increase in the integrins-VEGFR2 interaction and the YAP/TAZ signaling pathway activation. In addition, pharmacological inhibition of integrins or YAP/TAZ signaling prevents the formation of AVMs as well as the endothelial polarization defects in *Acvrl1* mutant mice.

All these studies demonstrate that BMP9/ALK1 signaling is essential to control LSS-induced EC quiescence and polarity *via* the VEGFR2-integrins complex and the activation of downstream signaling pathways, such as PI3K/AKT and YAP/TAZ.

#### JP-HHT: *SMAD4* mutation

In the mouse model of JP-HHT, postnatal endothelial *Smad4* deletion induces retinal AVMs and hypersprouting, as observed in the *Acvrl1* and *Eng* mutant mice (Crist et al., 2018; Ola et al., 2018). However, the authors found a vascular outgrowth defect that was also found in the HHT1 models but not in the HHT2 model (Crist et al., 2018). Similar to *Acvrl1* and *Eng* deletion, AVMs developed near the optic nerve where blood flow is high, and *Smad4* AVMs originate from defective venous ECs (Lee et al., 2021). In line with its upstream effectors, *Smad4* deletion also increased EC proliferation and PI3K/AKT signaling and inhibited EC migration against the flow, suggesting a similar mechanism in SMAD4 AVMs. Indeed, the inhibition of the BMP9/ALK1/SMAD4 signaling pathway promotes excessive AKT signaling, which inhibits the transcription factor Forkhead box protein O1 (FOXO1) and increases the expression of the proto-oncogene c-MYC (Ola et al., 2018). Ola et al. (2018) proposed that SMAD4 activation regulates Casein Kinase 2 (CK2) expression, which is responsible for PTEN phosphorylation and inhibition. Moreover, CK2 inhibition prevents PI3K/AKT overactivation and the formation of AVMs in *Smad4* mutant mice. These data suggest that the BMP9/ALK1/SMAD4 signaling pathway is essential in venous EC to prevent abnormal PI3K/AKT signaling, proliferation, and LSS-induced migration defect.

In line with these observations, knockdown of receptor-regulated SMADs (R-SMADs) SMAD1 and SMAD5 has also been shown to result in the formation of AVMs. Benn and colleagues reported that endothelial-specific simultaneous deletion of *Smad1* and *Smad5* resulted in the formation of retinal AVMs in areas with high blood flow, while reduced vessel regression and increased loop formation were observed in areas of lower blood flow (Benn et al., 2020). The formation of vascular shunts was also observed in yolk sacs of endothelial-specific *Smad1/5* knockout embryos at E9.25, which occurred in part as a consequence of decreased *Cx37* expression, leading to vessel enlargement and the formation of shunts in vessel segments of higher flow (Peacock et al., 2020). Altogether, these studies further highlight the crucial role of SMAD signaling in preventing AVM development.

Constitutive *Smad4* deletion in brain ECs increased EC proliferation, leading to cerebral hemorrhages (Li et al., 2011). In addition, the authors observed a decrease in NOTCH expression, suggesting a connection between the NOTCH and ALK1/SMAD4 pathways in the maintenance of brain vascular integrity. Interestingly, mutant mouse embryos for NOTCH effectors, such as delta-like-4 (*Dll4)* and Recombination Signal Binding Protein For Immunoglobulin Kappa J Region (*Rbpj)*, exhibit bAVMs (Krebs et al., 2004). The involvement of this pathway in bAVM formation is discussed in a later section.

#### HHT5-BMP9/10

BMP9 and BMP10 signaling have redundant functions in mice during postnatal life, and both ligands have to be inhibited to phenocopy *Acvrl1* and *Eng* mutant mice. Indeed, the injection of BMP9 and BMP10 blocking antibodies in neonatal pups induces retinal AVMs near the optic nerve, as well as hypersprouting and hyperplasia (Baeyens et al., 2016; Ruiz et al., 2016). Moreover, BMP9 inhibition is sufficient to increase PI3K/AKT signaling and proliferation of EC under-flow *in vitro*. Similar to other HHT models, inhibition of the PI3K/AKT or VEGFR2 pathway prevents and rescues EC proliferation and AVMs formation induced by BMP9 and BMP10 blockade (Baeyens et al., 2016; Ola et al., 2016).

Interestingly, sirolimus, an inhibitor of AKT downstream effector mammalian target of rapamycin (mTOR) pathway, can rescue SMAD1/5/8 signaling and AVMs by activating ALK2 in BMP9/10 immunodepleted mice (Ruiz et al., 2020).

**FIGURE 2:**
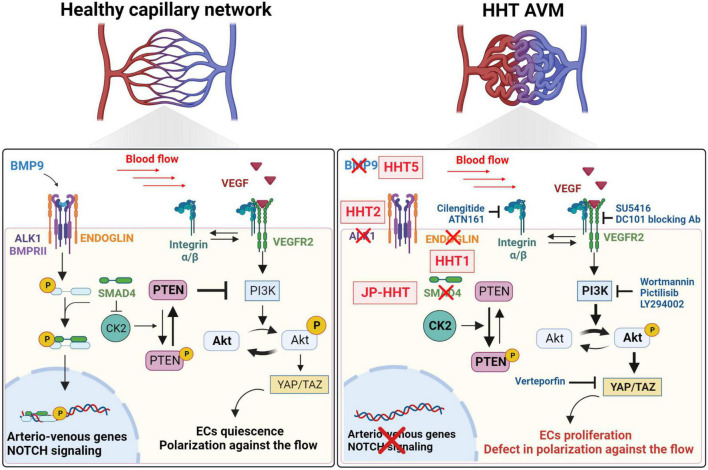
Signaling pathways known to be involved in the pathogenesis of AVMs. In healthy capillary networks, blood flow potentiates BMP/ALK1 signaling pathway in ECs to regulate the expression of genes involved in arterio-venous specification and NOTCH signaling pathway. BMP/ALK1/SMAD4 pathway will inhibit blood flow- and VEGFR2-induced PI3K/AKT signaling. Indeed SMAD4 inhibits the expression of CK2, which allows the phosphorylation, and inhibition of PTEN, a negative regulator of the PI3K/AKT signaling, which will promote ECs quiescence and polarization against the flow. HHT1, HHT2, JP-HHT, and HHT5 are induced by *ENG*, *ACVRL1* (ALK1), *SMAD4*, and *GDF2* (BMP9) heterozygous mutations, respectively. CK2 inhibition is removed and its expression increases leading to an enhanced phosphorylation of PTEN which will not be able to inhibit PI3K/AKT signaling induced by blood flow and VEGFR2. Increased AKT phosphorylation leads to higher YAP/TAZ expression and activity, hyperproliferation of ECs and migration defects against the flow.

Despite the specificities of distinct HHT types, the different mouse models suggest that there is one common mechanism that controls AVM formation. The loss of the BMP/ALK1/SMAD4 signaling pathway in venous ECs leads to an abnormal response to blood flow, including the increase of PI3K/AKT signaling activation and EC proliferation and inhibition of LSS-induced EC polarity and migration against the flow. Then, mutant ECs accumulate in abnormal capillaries, which get enlarged, experience higher blood flow, and acquire venous markers. Whereas arterial ECs might be dispensable, the decrease of NOTCH signaling and arterial marker expression are associated with AVMs. Ultimately, the loss of capillary bed and distinct EC phenotypes forms fragile AVMs (Figure 2). Further studies are required to investigate whether this mechanism is conserved in other tissues and humans.

## NOTCH signaling and brain AVMs

The NOTCH pathway is a highly conserved cell signaling system involved in cell fate decisions during several developmental processes, including cardiovascular development and neurogenesis. Mammalian cells possess four specific transmembrane NOTCH receptors, NOTCH1, NOTCH2, NOTCH3, and NOTCH4 (Gordon et al., 2008). NOTCH ligands are also transmembrane proteins that are members of the Delta-like and Jagged families in mammals: delta-like-1 (DLL1), DLL3, and DLL4, JAGGED-1, and JAGGED-2 (D’Souza et al., 2008). Upon activation by their specific ligands, NOTCH receptors undergo proteolytic processing, releasing the NOTCH intracellular domain (NICD), which translocates to the nucleus, where it can form a complex with the DNA-binding protein RBP-J to initiate transcription of its downstream targets, such as members of the Hairy and enhancer-of-split (HES), and Hairy and enhancer-of-split-related (HEY, HESR, HRT, or CHF) gene families (Castel et al., 2013). NOTCH signaling plays a critical role during vascular morphogenesis, as demonstrated by several studies in which deletion of genes coding for NOTCH receptors, ligands, or downstream effectors results in severe angiogenic defects (Swiatek et al., 1994; Hamada et al., 1999; Xue et al., 1999; Krebs et al., 2000; Domenga et al., 2004; Duarte et al., 2004). Specifically loss of NOTCH signaling has been associated with impaired tip/stalk EC specification in several *in vitro* and animal models (Noguera-Troise et al., 2006; Hellström et al., 2007; Leslie et al., 2007; Suchting et al., 2007). In addition to its roles in sprouting angiogenesis, NOTCH signaling has also been implicated in the specification of artery/vein EC. Indeed, studies have shown that NOTCH signaling induces the expression of several arterial markers and can suppress the expression of venous markers in developing blood vessels (Lawson et al., 2001; Iso et al., 2006; Trindade et al., 2008). As such, both gain-of-function and loss-of-function in NOTCH have resulted in abnormal arterial and venous specification (Lawson et al., 2001; Duarte et al., 2004; Trindade et al., 2008).

The abnormal arteriovenous specification has in several cases been associated with the development of arteriovenous shunts, suggesting a role for NOTCH signaling in the pathophysiology of AVMs. Interestingly, elevated NOTCH signaling has been found in human patients with idiopathic AVMs (ZhuGe et al., 2009, 2013; Yao et al., 2013; Li et al., 2014). Moreover, AVMs have been found in both Notch gain- and loss-of-function animal models, indicating that NOTCH signaling must be tightly regulated in ECs to regulate proper vascular morphogenesis. In mice, the expression of a constitutively active NOTCH4 results in AVMs in the liver, uterus, skin, and brain (Murphy et al., 2008, 2014). Similarly, EC-specific, constitutively active NOTCH1 also results in AVM formation during embryonic development, suggesting that increased activity of either Notch receptor is sufficient to cause brain AVMs (Krebs et al., 2010). DLL4 overexpression models have also been shown to be prone to AVM formation (Trindade et al., 2008). Conversely, decreased NOTCH signaling has also been associated with the development of AVM lesions. In zebrafish embryos, reduction of NOTCH signaling results in the formation of AVMs (Lawson et al., 2001), while in mouse embryos, both *Dll4* ± and *Rbpj* null embryos exhibit the presence of AVMs (Krebs et al., 2004). Endothelial-specific deletion of *Rbpj* from birth also results in abnormal AV shunting and tortuous vessels in the brain, intestine, and heart of P14 mice (Nielsen et al., 2014).

Several studies have documented the crosstalk between endothelial BMP/ALK1/SMAD and NOTCH signaling pathways during vascular development (Larrivée et al., 2012; Moya et al., 2012; Ricard et al., 2012). Functionally, it was observed that BMP9 stimulation could counteract the effects of NOTCH inhibition using *in vitro* angiogenesis assays as well as mouse models of vascular development (Larrivée et al., 2012). It was also demonstrated that NOTCH target genes are decreased in *Eng*-deficient cells, but that ALK1 overexpression could rescue their expression confirming the regulatory effects of ALK1 on NOTCH signaling (Hwan Kim et al., 2020). Interestingly, activated SMADs can co-immunoprecipitate with NICD to potentiate HEY1 expression, suggesting a direct interaction between components of these signaling pathways (Itoh et al., 2004). Finally, similarly to flow-dependent activation of ALK1 (Baeyens et al., 2016), LSS at an arterial magnitude also activates NOTCH signaling to control arterial specification and ECs quiescence (Fang et al., 2017). These insights suggest that mechanical forces may also contribute to the crosstalk between ALK1 and NOTCH signaling. Taken together, these studies demonstrate that ALK1/BMP/SMAD signaling is an important modulator of NOTCH signaling in ECs.

In contrast to several models of idiopathic AVMs, which display increased NOTCH signaling, AVMs in HHT models tend to display decreased NOTCH signaling. In *Acvrl1* knockout mouse models, the development of AVMs has been associated with reduced *Notch1* and *Jag1* expression (Tual-Chalot et al., 2014). Other components of BMP signaling, which directly affect ALK1 signaling and lead to AVM formation, have also been shown to have decreased Notch signaling. For example, deletion of *Mgp*, which can act as a regulatory protein for BMPs, causes AVM formation in multiple organs partly by modulating *Acvrl1* expression. *Mgp* depletion in ECs upregulated *Acvrl1* expression and, in turn, increased NOTCH signaling in an ALK1-dependent manner (Yao et al., 2011, 2013). On the other hand, other studies did not report alterations in NOTCH signaling following impairment of ALK1 or ENG signaling. In zebrafish, Rochon et al. (2015) showed that AVMs in *acvrl1* mutants arose independently of perturbations in NOTCH signaling, suggesting that it may not be critical for AVM formation in this context. Therefore, the role of NOTCH signaling in HHT pathophysiology still remains unclear, and further evaluations of NOTCH signaling components and downstream targets in AVMs from HHT patients will be needed to offer additional insights into its role in the pathogenesis of these lesions.

## Sporadic brain AVMs and RAS signaling

bAVMs found in HHT patients are a familial form with well identified mutations. However, most cases of bAVMs are sporadic, involving somatic mutations mainly associated with RAS signaling, and mechanisms are not well understood.

The MAPK/ERK signaling pathway is also involved in controlling EC proliferation and arteriovenous specification during development. Growth factor receptors, such as EGFR2 or VEGFR2, activate proto-oncogene Rat sarcoma virus proteins (RAS; HRAS, KRAS, and NRAS in humans), which triggers the downstream MAPK/ERK chain, including Rapidly Accelerated Fibrosarcoma (RAF) and Mitogen-activated protein kinase kinase (MEK). Finally, the activated ERK will translocate into the nucleus to regulate the expression of genes involved in cell proliferation (Karar and Maity, 2011; Tan et al., 2013; Simons et al., 2016).

Somatic mutations in the MAPK pathway lead to AVMs formation, such as activating mutations of KRAS, BRAF, or MAPK1/2. Moreover, loss-of-function mutations of RASA1, a negative regulator of RAS signaling, are associated with capillary-malformation arteriovenous malformation (CM-AVM1) and bAVMs in humans (Eerola et al., 2003; Revencu et al., 2008). These AVMs are all characterized by increased ERK phosphorylation and cell proliferation (Eerola et al., 2003; Boon et al., 2005; Couto et al., 2017; Hong et al., 2019).

In mice, overexpression of KRAS in brain ECs specifically leads to the formation of bAVMs, confirming the importance of ECs in the pathogenesis of AVMs (Fish et al., 2020; Park H. et al., 2021). Furthermore, overexpression of KRAS in zebrafish embryos leads to AVMs and increased activity of proteins involved in angiogenesis, such as DLL4 (Al-Olabi et al., 2018; Fish et al., 2020). Interestingly, overexpression of KRAS in mouse cerebral ECs results in overexpression of VEGFR2 expression (Park E. S. et al., 2021). Surprisingly, while *Rasa1* loss of function mice exhibit vascular and lymphatic defects, they did not feature bAVMs (Henkemeyer et al., 1995; Lapinski et al., 2012, 2017; Lubeck et al., 2014; Chen et al., 2019). MEK inhibition decreases the number of shunts induced by KRAS overexpression in brain ECs. Interestingly, inhibition of the PI3K/AKT pathway has no effect (Fish et al., 2020). Therefore, while EC proliferation is a common feature with HHT AVM, mechanisms might differ regarding EC polarity and migration against the flow.

## Perivascular cells contribution to brain AVMs

The various HHT models developed in recent years have demonstrated the importance of the endothelial BMP/ALK1 pathway in the pathogenesis of AVMs. However, PCs are also essential for vascular integrity and morphogenesis (Armulik et al., 2011). To control capillary coverage by PCs, ECs express soluble Platelet-derived growth factor B (PDGFB), which activates the PDGF receptor (PDGFR) at the PC membrane. PDGFB deletion induces PC loss and micro aneurysms (Lindahl et al., 1997; Hellström et al., 2001; Lindblom et al., 2003). While AVMs are associated with decreased PC coverage in mice and humans (Chen W. et al., 2013; Winkler et al., 2018), the PC contribution to HHT AVMs remains unclear.

It has been shown that following *Acvrl1* deletion, there was a decrease in the expression of PDGFRB, but not PDGFB, which could explain the defect in PC recruitment (Chen W. et al., 2013). Thalidomide had already been shown to inhibit angiogenesis. The injection of thalidomide, a drug that reduced the severity and frequency of epistaxis in HHT patients, into mice depleted for *Eng* prevented AVMs-induced bleeding and excessive angiogenesis. Interestingly, it also restores PC coverage by increasing PDGFRB levels (Lebrin et al., 2010; Zhu et al., 2018).

These data suggest that PCs may be involved in the onset or progression of AVMs. Recently, it was shown that a PC-specific deletion of *Rbpj* was sufficient to induce the formation of retinal AVMs. Interestingly, the deletion of *Rbpj* in SMC leads to the formation of AVMs in the retina (Nadeem et al., 2020). It was also reported that *Notch1* ±; *Notch3*-/- mice developed AVMs in the retina (Kofler et al., 2015). Notch3 deficiency in this model compromised PC function, which in turn exacerbated EC activation caused by Notch1 haploinsufficiency.

vSMCs are cells found in arteries and veins, and this coverage is also reduced in bAVMs. However, their involvement in the formation of AVMs is controversial. Indeed, in 2014 a study compared the effect of *Acvrl1* and *Eng* deletion in ECs and SMCs using *Scl*-CreER and *Myh11*-CreER driver mice (Table 1). The authors showed that only endothelial deletion allowed AVMs formation in a model of injury-induced skin AVMs (Garrido-Martin et al., 2014). However, a recent study showed that deletion of *Acvrl1* in SMCs using *Tagln*-Cre mice induced the formation of bAVMs associated with a better survival of the mice that allows the monitoring of bAVMs formation over time, and to have a pathological model more representative of the patients. However, *Tagln*-CRE can be expressed by some ECs which could be sufficient to induce AVMs. It is therefore possible that *Acvrl1* mosaic deletion in ECs would be the primary cause of these bAVMs over time rather than the deletion in SMCs (Han et al., 2021). Further studies are needed to understand the exact role of SMCs and PCs in developing bAVMs.

## Therapeutic perspectives

Currently, treatment options for bAVMs are limited and only based on surgeries: surgical resection, endovascular embolization, and/or stereotactic radiosurgery. Knowing the risk of intracranial hemorrhage (ICH) (which varies from 0.9 to 34.3%), the treatment of unruptured bAVMs has become controversial, as untreated patients’ fate may be less morbid than those treated with invasive therapies. Thus, the demand for pharmacologic treatment options is high (Chen W. et al., 2014; Shaligram et al., 2019).

Because of the enhanced angiogenesis associated with bAVMs formation, the most common pharmacologic target is VEGF. The anti-VEGF drug bevacizumab is currently undergoing phase III clinical trial for HHT (NCT 03227263) after showing promising effects on other HHT symptoms such as epistaxis and gastrointestinal bleedings in phase II clinical trials and case reports (Oosting et al., 2009), without showing adverse effects. Altogether, these results suggest that anti-VEGF therapies could represent good alternatives or complements to surgical procedures (Chen W. et al., 2014; Epperla and Hocking, 2015; Snodgrass et al., 2021).

Elucidation of the signaling events associated with HHT has confirmed the contribution of inadequate BMP9/ALK1/SMAD signaling in the formation of bAVMs, and as such, some research was done using tacrolimus, an anti-inflammatory FDA-approved drug, to evaluate whether it could potentiate the activity of the impaired BMP9/ALK1/SMAD pathway present in HHT patients resulting from haploinsufficiency. Indeed, a preclinical study demonstrated that tacrolimus could reduce the development of retinal AVMs in BMP9/10 immunodepleted mice, and it is currently undergoing a clinical trial to evaluate its ability to reduce epistaxis (NCT 03152019) (Robert et al., 2020; Snodgrass et al., 2021).

A better understanding of the signaling pathways inducing bAVMs opens up opportunities for pharmacological treatments using other drugs such as tyrosine kinase inhibitors. Some case reports already showed an improvement of epistaxis and telangiectasia in mostly HHT1 and two patients with different tyrosine kinase inhibitors (Robert et al., 2020; Snodgrass et al., 2021) targeting the VEGFR2 and the ANGPT2 receptor TIE2, which are overactivated in HHT. Thus, two clinical trials are beginning to test the impact of pazopanib, a selective multi-targeted receptor tyrosine kinase inhibitor, on epistaxis and telangiectasia (NCT03850964/NCT03850964). However, preclinical studies in mouse models showed no improvement in wound-induced skin AVMs, leaving little hope for a potential rescue of bAVMs (Robert et al., 2020; Snodgrass et al., 2021).

Other potential treatments are based on inhibiting the PI3K/AKT/mTOR pathway, which is highly activated downstream of ANGPT2 and VEGF signaling. The most common mTOR inhibitor is sirolimus (rapamycin), which was shown to prevent bleeding and anemia in preclinical mouse models of HHT by controlling VEGFR2 and mTOR overactivation (Robert et al., 2020). One case report and a clinical study suggested that sirolimus could control and rescue HHT-induced bleedings and vascular malformations (Skaro et al., 2006; Dupuis-Girod et al., 2010). Apart from sirolimus, using PI3K inhibitors that are already FDA-approved for chemotherapy could also represent an excellent potential therapeutic avenue.

Altogether, although preclinical and clinical tests on pharmacological treatments are ongoing, the probability of reversing the bAVMs is still weak. Despite advances in research to identify the mechanisms, surgical treatments are still the best option to treat bAVMs. Thus, improving surgical conditions and tools currently remains the optimal avenue to improve the survival of HHT patients (Chen W. et al., 2014; Shaligram et al., 2019; Robert et al., 2020).

## Conclusion

Identifying the molecular mechanisms associated with bAVM formation is an essential step toward elucidating the mechanisms of these malformations. Several additional questions, however, remain to be addressed. Are there modifying genes that may explain the clinical variability of bAVMs? How does inadequate ALK1/ENG signaling affect the response of ECs to mechanical stress, and how does this contribute to bAVM formation? Is PI3K/AKT signaling increased in HHT bAVMs, and could it be targeted to improve the malformations? What is the contribution of NOTCH signaling in the formation of bAVMs in HHT patients? In addition to these questions, one significant challenge remains to characterize and understand the common mechanisms underlying idiopathic bAVMs and those observed in HHT patients. The recent identification of several signaling pathways involved in bAVMs formation, as well as the analysis of several experimental animal models, have provided several clues to this goal and could pave the way for developing novel therapies.

## Author contributions

ED, TA, BL, and AD wrote the manuscript, collected literature information, and edited the manuscript. All authors reviewed and commented on the manuscript.

## Abbreviations

ACVRL1: Activin A receptor like type 1
AVF: Arteriovenous fistulas
AVM: Arteriovenous malformations
bAVM: Brain AVM
BBB: Blood-brain-barrier
BMP: Bone Morphogenetic Protein
CK2: Casein Kinase 2
CNS: Central nervous system
CVM: Capillary vascular malformation
EC: Endothelial cell
ENG: Endoglin
HHT: Hereditary hemorrhagic telangiectasia
JP-HHT: Juvenile polyposis-HHT
LSS: Laminar Shear Stress
NVU: Neurovascular unit
PC: Pericyte
PDGF: Platelet-derived growth factor B
PDGFR: Platelet-derived growth factor B receptor
PNVP: Perineural vascular plexus
SMC: Smooth muscle cells
TGFβ: Transforming growth factor β
VEGF: Vascular endothelial growth factor
VEGFR: Vascular endothelial growth factor receptor.

## References

[B1] Abecassis I. J. Xu D. S. Batjer H. H. Bendok B. R. (2014). Natural history of brain arteriovenous malformations: A systematic review. *Neurosurg. Focus* 37:E7. 10.3171/2014.6.FOCUS14250 25175445

[B2] Adams R. H. Alitalo K. (2007). Molecular regulation of angiogenesis and lymphangiogenesis. *Nat. Rev. Mol. Cell Biol.* 8 464–478. 10.1038/nrm2183 17522591

[B3] Akimoto S. Mitsumata M. Sasaguri T. Yoshida Y. (2000). Laminar shear stress inhibits vascular endothelial cell proliferation by inducing cyclin-dependent kinase inhibitor p21Sdi1/Cip1/Waf1. *Circ. Res.* 86 185–90. 10.1161/01.RES.86.2.185 10666414

[B4] Allinson K. R. Carvalho R. L. van den Brink S. Mummery C. L. Arthur H. M. (2007). Generation of a floxed allele of the mouse endoglin gene. *Genesis* 45 391–95. 10.1002/dvg.20284 17506087 PMC2077828

[B5] Al-Olabi L. Polubothu S. Dowsett K. Andrews K. A. Stadnik P. Joseph A. P. (2018). Mosaic RAS/MAPK variants cause sporadic vascular malformations which respond to targeted therapy. *J. Clin. Investig.* 128 1496–1508. 10.1172/JCI98589 29461977 PMC5873857

[B6] Alsina-Sanchís E. García-Ibáñez Y. Figueiredo A. M. Riera-Domingo C. Figueras A. Matias-Guiu X. (2018). ALK1 loss results in vascular hyperplasia in mice and humans through PI3K activation. *Arterioscler. Thromb. Vasc. Biol.* 38 1216–29. 10.1161/ATVBAHA.118.310760 29449337

[B7] Alt A. Miguel-Romero L. Donderis J. Aristorena M. Blanco F. J. Round A. (2012). Structural and functional insights into endoglin ligand recognition and binding. *PLoS One* 7:12. 10.1371/journal.pone.0029948 22347366 PMC3275592

[B8] Armulik A. Genové G. Betsholtz C. (2011). Pericytes: Developmental, physiological, and pathological perspectives, problems, and promises. *Dev. Cell* 21 193–215. 10.1016/j.devcel.2011.07.001 21839917

[B9] Arthur H. M. Ure J. Smith A. J. Renforth G. Wilson D. I. Torsney E. (2000). Endoglin, an ancillary TGFβ receptor, Is required for extraembryonic angiogenesis and plays a key role in heart development. *Dev. Biol.* 217 42–53. 10.1006/dbio.1999.9534 10625534

[B10] Baeyens N. Larrivée B. Ola R. Hayward-Piatkowskyi B. Dubrac A. Huang B. (2016). Defective fluid shear stress mechanotransduction mediates hereditary hemorrhagic telangiectasia. *J. Cell Biol.* 214 807–16. 10.1083/jcb.201603106 27646277 PMC5037412

[B11] Bautch V. L. James J. M. (2009). Neurovascular development. *Cell Adhes. Migr.* 3 199–204. 10.4161/cam.3.2.8397 19363295 PMC2679887

[B12] Bayrak-Toydemir P. McDonald J. Markewitz B. Lewin S. Miller F. Chou L. S. (2006). Genotype-phenotype correlation in hereditary hemorrhagic telangiectasia: Mutations and manifestations. *Am. J. Med. Genet. Part A* 140 463–70. 10.1002/ajmg.a.31101 16470787

[B13] Benn A. Alonso F. Mangelschots J. Génot E. Lox M. Zwijsen A. (2020). BMP-SMAD1/5 Signaling regulates retinal vascular development. *Biomolecules* 10:488. 10.3390/biom10030488 32210087 PMC7175193

[B14] Bernabeu M. O. Jones M. L. Nielsen J. H. Krüger T. Nash R. W. Groen D. (2014). Computer simulations reveal complex distribution of haemodynamic forces in a mouse retina model of angiogenesis. *J. R. Soc. Interface* 11:20140543. 10.1098/rsif.2014.0543 25079871 PMC4233731

[B15] Bidart M. Ricard N. Levet S. Samson M. Mallet C. David L. (2012). BMP9 Is produced by hepatocytes and circulates mainly in an active mature form complexed to its prodomain. *Cell. Mol. Life Sci.* 69 313–24. 10.1007/s00018-011-0751-1 21710321 PMC11114909

[B16] Bideau A. Plauchu H. Brunet G. Robert J. (1989). Epidemiological investigation of rendu-osler disease in France: Its geographical distribution and prevalence. *Popul. Engl. Sel.* 44 3–22. 12157905

[B17] Biswas S. Cottarelli A. Agalliu D. (2020). Neuronal and glial regulation of CNS angiogenesis and barriergenesis. *Development* 147:dev182279. 10.1242/dev.182279 32358096 PMC7197727

[B18] Boon L. M. Mulliken J. B. Vikkula M. (2005). RASA1: Variable phenotype with capillary and arteriovenous malformations. *Curr. Opin. Genet. Dev. Genet. Dis.* 15 265–69. 10.1016/j.gde.2005.03.004 15917201

[B19] Bourdeau A. Dumont D. J. Letarte M. (1999). A murine model of hereditary hemorrhagic telangiectasia. *J. Clin. Investig.* 104 1343–51. 10562296 10.1172/JCI8088 PMC409846

[B20] Bouvard C. Tu L. Rossi M. Desroches-Castan A. Berrebeh N. Helfer E. (2021). Different cardiovascular and pulmonary phenotypes for single- and double-knock-out mice deficient in BMP9 and BMP10. *Cardiovasc. Res.* 118 1805–20. 10.1093/cvr/cvab187 34086873 PMC9215199

[B21] Brinjikji W. Iyer V. N. Lanzino G. Thielen K. R. Wood C. P. (2017). Natural history of brain capillary vascular malformations in hereditary hemorrhagic telangiectasia patients. *J. Neurointerv. Surg.* 9 26–28. 10.1136/neurintsurg-2015-012252 26919971

[B22] Brown R. D. Wiebers D. O. Torner J. C. O’Fallon W. M. (1996). Frequency of intracranial hemorrhage as a presenting symptom and subtype analysis: A population-based study of intracranial vascular malformations in Olmsted County, Minnesota. *J. Neurosurg.* 85 29–32. 10.3171/jns.1996.85.1.0029 8683279

[B23] Capasso T. L. Li B. Volek H. J. Khalid W. Rochon E. R. Anbalagan A. (2020). BMP10-mediated ALK1 signaling is continuously required for vascular development and maintenance. *Angiogenesis* 23 203–20. 10.1007/s10456-019-09701-0 31828546 PMC7165044

[B24] Castel D. Mourikis P. Bartels S. J. Brinkman A. B. Tajbakhsh S. Stunnenberg H. G. (2013). Dynamic binding of RBPJ is determined by notch signaling status. *Genes Dev.* 27 1059–71. 10.1101/gad.211912.112 23651858 PMC3656323

[B25] Chen D. Teng J. M. North P. E. Lapinski P. E. King P. D. (2019). RASA1-Dependent cellular export of collagen IV controls blood and lymphatic vascular development. *J. Clin. Investig.* 129 3545–61. 10.1172/JCI124917 31185000 PMC6715364

[B26] Chen H. Brady Ridgway J. Sai T. Lai J. Warming S. Chen H. (2013). Context-dependent signaling defines roles of BMP9 and BMP10 in embryonic and postnatal development. *Proc. Natl. Acad. Sci. U.S.A.* 110 11887–92. 10.1073/pnas.1306074110 23812757 PMC3718114

[B27] Chen W. Choi E. J. McDougall C. M. Su H. (2014). Brain arteriovenous malformation modeling, pathogenesis, and novel therapeutic targets. *Transl. Stroke Res.* 5 316–29. 10.1007/s12975-014-0343-0 24723256 PMC4081044

[B28] Chen W. Guo Y. Walker E. J. Shen F. Jun K. Oh S. P. (2013). Reduced mural cell coverage and impaired vessel integrity after angiogenic stimulation in the Alk1-deficient brain. *Arterioscler. Thromb. Vasc. Biol* 33 305–10. 10.1161/ATVBAHA.112.300485 23241407 PMC3569037

[B29] Chen Y. G. Massagué J. (1999). Smad1 recognition and activation by the ALK1 group of transforming growth factor-β family receptors. *J. Biol. Chem.* 274 3672–77. 10.1074/jbc.274.6.3672 9920917

[B30] Choi E. J. Chen W. Jun K. Arthur H. M. Young W. L. Su H. (2014). Novel brain arteriovenous malformation mouse models for type 1 hereditary hemorrhagic telangiectasia. *PLoS One* 9:e88511. 10.1371/journal.pone.0088511 24520391 PMC3919779

[B31] Choi E. J. Walker E. J. Shen F. Oh S. P. Arthur H. M. Young W. L. (2012). Minimal homozygous endothelial deletion of eng with VEGF stimulation is sufficient to cause cerebrovascular dysplasia in the adult mouse. *Cerebrovasc. Dis.* 33 540–47. 10.1159/000337762 22571958 PMC3569027

[B32] Coelho-Santos V. Shih A. Y. (2020). Postnatal development of cerebrovascular structure and the neurogliovascular unit. *Wiley Interdiscip. Rev. Dev. Biol.* 9:e363. 10.1002/wdev.363 31576670 PMC7027551

[B33] Cole S. G. Begbie M. E. Wallace G. M. F. Shovlin C. L. (2005). A new locus for Hereditary haemorrhagic telangiectasia (HHT3) maps to chromosome 5. *J. Med. Genet.* 42 577–82. 10.1136/jmg.2004.028712 15994879 PMC1736109

[B34] Coon B. G. Baeyens N. Han J. Budatha M. Ross T. D. Fang J. S. (2015). Intramembrane binding of VE-cadherin to VEGFR2 and VEGFR3 assembles the endothelial mechanosensory complex. *J. Cell Biol.* 208 975–86. 10.1083/jcb.201408103 25800053 PMC4384728

[B35] Corti P. Young S. Chen C. Y. Patrick M. J. Rochon E. R. Pekkan K. (2011). Interaction between Alk1 and blood flow in the development of arteriovenous malformations. *Development* 138 1573–82. 10.1242/dev.060467 21389051 PMC3062425

[B36] Couto J. A. Huang A. Y. Konczyk D. J. Goss J. A. Fishman S. J. Mulliken J. B. (2017). Somatic MAP2K1 mutations are associated with extracranial arteriovenous malformation. *Am. J. Hum. Genet.* 100 546–54. 10.1016/j.ajhg.2017.01.018 28190454 PMC5339083

[B37] Crist A. M. Lee A. R. Patel N. R. Westhoff D. E. Meadows S. M. (2018). Vascular deficiency of Smad4 causes arteriovenous malformations: A mouse model of hereditary hemorrhagic telangiectasia. *Angiogenesis* 21 363–80. 10.1007/s10456-018-9602-0 29460088 PMC5878194

[B38] Daneman R. Prat A. (2015). The blood–brain barrier. *Cold Spring Harb. Perspect. Biol.* 7:a020412. 10.1101/cshperspect.a020412 25561720 PMC4292164

[B39] David L. Mallet C. Keramidas M. Lamandé N. Gasc J. M. Dupuis-Girod S. (2008). Bone morphogenetic protein-9 is a circulating vascular quiescence factor. *Circ. Res.* 102 914–22. 10.1161/CIRCRESAHA.107.165530 18309101 PMC2561062

[B40] David L. Mallet C. Mazerbourg S. Feige J. J. Bailly S. (2006). Identification of BMP9 and BMP10 as functional activators of the orphan activin receptor-like kinase 1 (ALK1) in endothelial cells. *Blood* 109 1953–61. 10.1182/blood-2006-07-034124 17068149

[B41] Domenga V. Fardoux P. Lacombe P. Monet M. Maciazek J. Krebs L. T. (2004). Notch3 Is required for arterial identity and maturation of vascular smooth muscle cells. *Genes Dev.* 18 2730–35. 10.1101/gad.308904 15545631 PMC528893

[B42] D’Souza B. Miyamoto A. Weinmaster G. (2008). The many facets of Notch ligands. *Oncogene* 27 5148–67. 10.1038/onc.2008.229 18758484 PMC2791526

[B43] Duarte A. Hirashima M. Benedito R. Trindade A. Diniz P. Bekman E. (2004). Dosage-sensitive requirement for mouse Dll4 in artery development. *Genes Dev.* 18 2474–78. 10.1101/gad.1239004 15466159 PMC529534

[B44] Dupuis-Girod S. Chesnais A. L. Ginon I. Dumortier J. Saurin J. C. Finet G. (2010). Long-term outcome of patients with hereditary hemorrhagic telangiectasia and severe hepatic involvement after orthotopic liver transplantation: A single-center study. *Liver Transplant.* 16 340–47. 10.1002/lt.21990 20209594

[B45] Eerola I. Boon L. M. Mulliken J. B. Burrows P. E. Dompmartin A. Watanabe S. (2003). Capillary malformation–arteriovenous malformation, a new clinical and genetic disorder caused by RASA1 mutations. *Am. J. Hum. Genet.* 73 1240–49. 10.1086/379793 14639529 PMC1180390

[B46] Epperla N. Hocking W. (2015). Blessing for the bleeder: Bevacizumab in hereditary hemorrhagic telangiectasia. *Clin. Med. Res.* 13 32–35. 10.3121/cmr.2013.1205 24667223 PMC4435085

[B47] Etienne-Manneville S. Hall A. (2001). Integrin-mediated activation of Cdc42 controls cell polarity in migrating astrocytes through PKCζ. *Cell* 106 489–98. 10.1016/S0092-8674(01)00471-8 11525734

[B48] Fang J. S. Coon B. G. Gillis N. Chen Z. Qiu J. Chittenden T. W. (2017). Shear-induced notch-Cx37-P27 axis arrests endothelial cell cycle to enable arterial specification. *Nat. Commun.* 8:2149. 10.1038/s41467-017-01742-7 29247167 PMC5732288

[B49] Fierstra J. Conklin J. Krings T. Slessarev M. Han J. S. Fisher J. A. (2011). Impaired Peri-Nidal Cerebrovascular Reserve in Seizure Patients with Brain Arteriovenous Malformations. *Brain* 134 100–109. 10.1093/brain/awq286 21109501

[B50] Fish J. E. Flores Suarez C. P. Boudreau E. Herman A. M. Gutierrez M. C. Gustafson D. (2020). Somatic gain of KRAS function in the endothelium is sufficient to cause vascular malformations that require MEK but not PI3K signaling. *Circ. Res.* 127 727–43. 10.1161/CIRCRESAHA.119.316500 32552404 PMC7447191

[B51] Fleetwood I. G. Steinberg G. K. (2002). Arteriovenous malformations. *Lancet* 359 863–73. 10.1016/S0140-6736(02)07946-1 11897302

[B52] Fonseca C. G. Barbacena P. Franco C. A. (2020). Endothelial cells on the move: Dynamics in vascular morphogenesis and disease. *Vasc. Biol.* 2:H29–43. 10.1530/VB-20-0007 32935077 PMC7487603

[B53] Gallione C. J. Repetto G. M. Legius E. Rustgi A. K. Schelley S. L. Tejpar S. (2004). A combined syndrome of juvenile polyposis and hereditary haemorrhagic telangiectasia associated with mutations in MADH4 (SMAD4). *Lancet* 363 852–59. 10.1016/S0140-6736(04)15732-2 15031030

[B54] Gariano R. F. Gardner T. W. (2005). Retinal angiogenesis in development and disease. *Nature* 438 960–66. 10.1038/nature04482 16355161

[B55] Garrido-Martin E. M. Nguyen H. L. Cunningham T. A. Choe S. W. Jiang Z. Arthur H. M. (2014). Common and distinctive pathogenetic features of arteriovenous malformations in hereditary hemorrhagic telangiectasia 1 and hereditary hemorrhagic telangiectasia 2 animal models—brief report. *Arterioscler. Thromb. Vasc. Biol.* 34 2232–36. 10.1161/ATVBAHA.114.303984 25082229

[B56] Gordon W. R. Arnett K. L. Blacklow S. C. (2008). The molecular logic of notch signaling – a structural and biochemical perspective. *J. Cell Sci.* 121 3109–19. 10.1242/jcs.035683 18799787 PMC2696053

[B57] Graupera M. Potente M. (2013). Regulation of angiogenesis by PI3K signaling networks. *Exp. Cell Res.* 319 1348–55. 10.1016/j.yexcr.2013.02.021 23500680

[B58] Griauzde J. Wilseck Z. M. Chaudhary N. Pandey A. S. Vercler C. J. Kasten S. J. (2020). Endovascular treatment of arteriovenous malformations of the head and neck: Focus on the yakes classification and outcomes. *J. Vasc. Int. Radiol.* 31 1810–16. 10.1016/j.jvir.2020.01.036 32958379

[B59] Gross B. A. Du R. (2013). Natural history of cerebral arteriovenous malformations: A meta-analysis: Clinical article. *J. Neurosurg.* 118 437–43. 10.3171/2012.10.JNS121280 23198804

[B60] Haddad-Tóvolli R. Dragano N. R. V. Ramalho A. F. S. Velloso L. A. (2017). Development and function of the blood-brain barrier in the context of metabolic control. *Front. Neurosci.* 11:224. 10.3389/fnins.2017.00224 28484368 PMC5399017

[B61] Haitjema T. Disch F. Overtoom T. T. Westermann C. J. Lammers J. W. (1995). Screening family members of patients with hereditary hemorrhagic telangiectasia. *Am. J. Med.* 99 519–24. 10.1016/S0002-9343(99)80229-0 7485210

[B62] Hamada Y. Kadokawa Y. Okabe M. Ikawa M. Coleman J. R. Tsujimoto Y. (1999). Mutation in ankyrin repeats of the mouse notch2 gene induces early embryonic lethality. *Development* 126 3415–24. 10.1242/dev.126.15.3415 10393120

[B63] Han C. Choe S.-W. Kim Y. H. Acharya A. P. Keselowsky B. G. Sorg B. S. (2014). VEGF neutralization can prevent and normalize arteriovenous malformations in an animal model for hereditary hemorrhagic telangiectasia 2. *Angiogenesis* 17 823–30. 10.1007/s10456-014-9436-3 24957885 PMC4177352

[B64] Han C. Lang M. J. Nguyen C. L. Melendez E. L. Mehta S. Turner G. H. (2021). Novel experimental model of brain arteriovenous malformations using conditional Alk1 gene deletion in transgenic mice. *J. Neurosurg.* 137 163–74. 10.3171/2021.6.JNS21717 34740197

[B65] Hellström M. Gerhardt H. Kalén M. Li X. Eriksson U. Wolburg H. (2001). Lack of pericytes leads to endothelial hyperplasia and abnormal vascular morphogenesis. *J. Cell Biol.* 153 543–54. 10.1083/jcb.153.3.543 11331305 PMC2190573

[B66] Hellström M. Phng L.-K. Hofmann J. J. Wallgard E. Coultas L. Lindblom P. (2007). Dll4 signalling through notch1 regulates formation of tip cells during angiogenesis. *Nature* 445 776–80. 10.1038/nature05571 17259973

[B67] Henkemeyer M. Rossi D. J. Holmyard D. P. Puri M. C. Mbamalu G. Harpal K. (1995). Vascular system defects and neuronal apoptosis in mice lacking ras GTPase-activating protein. *Nature* 377 695–701. 10.1038/377695a0 7477259

[B68] Hernandez F. Huether R. Carter L. Johnston T. Thompson J. Gossage J. R. (2015). Mutations in RASA1 and GDF2 identified in patients with clinical features of hereditary hemorrhagic telangiectasia. *Hum. Genome Var.* 2:15040. 10.1038/hgv.2015.40 27081547 PMC4785548

[B69] Hogan K. A. Ambler C. A. Chapman D. L. Bautch V. L. (2004). The neural tube patterns vessels developmentally using the VEGF signaling pathway. *Development* 131 1503–13. 10.1242/dev.01039 14998923

[B70] Hoh B. L. Chapman P. H. Loeffler J. S. Carter B. S. Ogilvy C. S. (2002). Results of multimodality treatment for 141 patients with brain arteriovenous malformations and seizures: Factors associated with seizure incidence and seizure outcomes. *Neurosurgery* 51 303–11. 12182768

[B71] Hong T. Yan Y. Li J. Radovanovic I. Ma X. Shao Y. W. (2019). High prevalence of KRAS/BRAF somatic mutations in brain and spinal cord arteriovenous malformations. *Brain* 142 23–34. 10.1093/brain/awy307 30544177

[B72] Howe J. R. Roth S. Ringold J. C. Summers R. W. Järvinen H. J. Sistonen P. I. (1998). Mutations in the SMAD4/DPC4 gene in juvenile polyposis. *Science* 280 1086–88. 10.1126/science.280.5366.1086 9582123

[B73] Hwan Kim Y. Vu P. N. Choe S. W. Jeon C. J. Arthur H. M. Vary C. P. H. (2020). Overexpression of activin receptor-like kinase 1 in endothelial cells suppresses development of arteriovenous malformations in mouse models of hereditary hemorrhagic telangiectasia. *Circ. Res.* 127 1122–37. 10.1161/CIRCRESAHA.119.316267 32762495 PMC7554133

[B74] Iso T. Maeno T. Oike Y. Yamazaki M. Doi H. Arai M. (2006). Dll4-selective notch signaling induces EphrinB2 gene expression in endothelial cells. *Biochem. Biophys. Res. Commun.* 341 708–14. 10.1016/j.bbrc.2006.01.020 16430858

[B75] Itoh F. Itoh S. Goumans M.-J. Valdimarsdottir G. Iso T. Dotto G. P. (2004). Synergy and antagonism between Notch and BMP receptor signaling pathways in endothelial cells. *EMBO J.* 23 541–51. 10.1038/sj.emboj.7600065 14739937 PMC1271801

[B76] Jin Y. Muhl L. Burmakin M. Wang Y. Duchez A. C. Betsholtz C. (2017). Endoglin prevents vascular malformation by regulating flow-induced cell migration and specification through VEGFR2 signalling. *Nat. Cell Biol.* 19 639–52. 10.1038/ncb3534 28530660 PMC5467724

[B77] Johnson D. W. Berg J. N. Baldwin M. A. Gallione C. J. Marondel I. Yoon S.-J. (1996). Mutations in the activin receptor–like kinase 1 gene in hereditary haemorrhagic telangiectasia type 2. *Nat. Genet.* 13 189–95. 10.1038/ng0696-189 8640225

[B78] Jonker L. Arthur H. M. (2002). Endoglin expression in early development is associated with vasculogenesis and angiogenesis. *Mech. Dev.* 110 193–96. 10.1016/S0925-4773(01)00562-7 11744382

[B79] Josephson C. B. Leach J.-P. Duncan R. Roberts R. C. Counsell C. E. Al-Shahi Salman R. (2011). Seizure risk from cavernous or arteriovenous malformations. *Neurology* 76 1548–54. 10.1212/WNL.0b013e3182190f37 21536634 PMC3100127

[B80] Kaplan L. Chow B. W. Gu C. (2020). Neuronal regulation of the blood–brain barrier and neurovascular coupling. *Nat. Rev. Neurosci.* 21 416–32. 10.1038/s41583-020-0322-2 32636528 PMC8934575

[B81] Karar J. Maity A. (2011). PI3K/AKT/mTOR pathway in angiogenesis. *Front. Mol. Neurosci.* 4:51. 10.3389/fnmol.2011.00051 22144946 PMC3228996

[B82] Kjeldsen A. D. Møller T. R. Brusgaard K. Vase P. Andersen P. E. (2005). Clinical symptoms according to genotype amongst patients with hereditary haemorrhagic telangiectasia. *J. Intern. Med.* 258 349–55. 10.1111/j.1365-2796.2005.01555.x 16164574

[B83] Kjeldsen A. D. Vase P. Green A. (1999). Hereditary haemorrhagic telangiectasia: A population-based study of prevalence and mortality in danish patients. *J. Intern. Med.* 245 31–39. 10.1046/j.1365-2796.1999.00398.x 10095814

[B84] Kofler N. M. Cuervo H. Uh M. K. Murtomäki A. Kitajewski J. (2015). Combined deficiency of Notch1 and Notch3 causes pericyte dysfunction, models CADASIL, and results in arteriovenous malformations. *Sci. Rep.* 5:16449. 10.1038/srep16449 26563570 PMC4643246

[B85] Komiyama M. Terada A. Ishiguro T. Watanabe Y. Nakajima H. Yamada O. (2015). Neuroradiological manifestations of hereditary hemorrhagic telangiectasia in 139 Japanese patients. *Neurol. Med. Chir.* 55 479–86. 10.2176/nmc.oa.2015-0040 26041630 PMC4628199

[B86] Krebs L. T. Shutter J. R. Tanigaki K. Honjo T. Stark K. L. Gridley T. (2004). Haploinsufficient lethality and formation of arteriovenous malformations in notch pathway mutants. *Genes Dev.* 18 2469–73. 10.1101/gad.1239204 15466160 PMC529533

[B87] Krebs L. T. Starling C. Chervonsky A. V. Gridley T. (2010). Notch1 activation in mice causes arteriovenous malformations phenocopied by ephrinB2 and EphB4 mutants. *Genesis* 48 146–50. 10.1002/dvg.20599 20101599 PMC2849749

[B88] Krebs L. T. Xue Y. Norton C. R. Shutter J. R. Maguire M. Sundberg J. P. (2000). Notch signaling is essential for vascular morphogenesis in mice. *Genes Dev.* 14 1343–52. 10.1101/gad.14.11.1343 10837027 PMC316662

[B89] Krings T. Kim H. Power S. Nelson J. Faughnan M. E. Young W. L. (2015). Neurovascular manifestations in hereditary hemorrhagic telangiectasia: Imaging features and genotype-phenotype correlations. *Am. J. Neuroradiol.* 36 863–70. 10.3174/ajnr.A4210 25572952 PMC4433843

[B90] Krings T. Ozanne A. Chng S. M. Alvarez H. Rodesch G. Lasjaunias P. L. (2005). Neurovascular phenotypes in hereditary haemorrhagic telangiectasia patients according to age. *Neuroradiology* 47 711–20. 10.1007/s00234-005-1390-8 16136265

[B91] Lan Y. Liu B. Yao H. Li F. Weng T. Yang G. (2007). Essential role of endothelial Smad4 in vascular remodeling and integrity. *Mol. Cell. Biol.* 27 7683–92. 10.1128/MCB.00577-07 17724086 PMC2169040

[B92] Langer D. J. Lasner T. M. Hurst R. W. Flamm E. S. Zager E. L. King J. T. (1998). Hypertension, small size, and deep venous drainage are associated with risk of hemorrhagic presentation of cerebral arteriovenous malformations. *Neurosurgery* 42 481–86. 10.1097/00006123-199803000-00008 9526981

[B93] Lapinski P. E. Kwon S. Lubeck B. A. Wilkinson J. E. Srinivasan R. S. Sevick-Muraca E. (2012). RASA1 maintains the lymphatic vasculature in a quiescent functional state in mice. *J. Clin. Investig.* 122 733–47. 10.1172/JCI46116 22232212 PMC3266774

[B94] Lapinski P. E. Lubeck B. A. Chen D. Doosti A. Zawieja S. D. Davis M. J. (2017). RASA1 regulates the function of lymphatic vessel valves in mice. *J. Clin. Investig.* 127 2569–85. 10.1172/JCI89607 28530642 PMC5490778

[B95] Larrivée B. Prahst C. Gordon E. del Toro R. Mathivet T. Duarte A. (2012). ALK1 signaling inhibits angiogenesis by cooperating with the notch pathway. *Dev. Cell* 22 489–500. 10.1016/j.devcel.2012.02.005 22421041 PMC4047762

[B96] Laux D. W. Young S. Donovan J. P. Mansfield C. J. Upton P. D. Roman B. L. (2013). Circulating Bmp10 Acts through endothelial Alk1 to mediate flow-dependent arterial quiescence. *Development* 140 3403–12. 10.1242/dev.095307 23863480 PMC3737721

[B97] Lawson N. D. Scheer N. Pham V. N. Kim C. H. Chitnis A. B. Campos-Ortega J. A. (2001). Notch signaling is required for arterial-venous differentiation during embryonic vascular development. *Development* 128 3675–83. 10.1242/dev.128.19.3675 11585794

[B98] Lebrin F. Srun S. Raymond K. Martin S. van den Brink S. Freitas C. (2010). Thalidomide stimulates vessel maturation and reduces epistaxis in individuals with hereditary hemorrhagic telangiectasia. *Nat. Med.* 16 420–28. 10.1038/nm.2131 20364125

[B99] Lee H.-W. Xu Y. He L. Choi W. Gonzalez D. Jin S.-W. (2021). Role of venous endothelial cells in developmental and pathologic angiogenesis. *Circulation* 144 1308–22. 10.1161/CIRCULATIONAHA.121.054071 34474596 PMC9153651

[B100] Leslie J. D. Ariza-McNaughton L. Bermange A. L. McAdow R. Johnson S. L. Lewis J. (2007). Endothelial signalling by the notch ligand delta-like 4 restricts angiogenesis. *Development* 134 839–44. 10.1242/dev.003244 17251261

[B101] Li D. Y. Sorensen L. K. Brooke B. S. Urness L. D. Davis E. C. Taylor D. G. (1999). Defective angiogenesis in mice lacking endoglin. *Science* 284 1534–37. 10.1126/science.284.5419.1534 10348742

[B102] Li F. Lan Y. Wang Y. Wang J. Yang G. Meng F. (2011). Endothelial Smad4 maintains cerebrovascular integrity by activating N-cadherin through cooperation with notch. *Dev. Cell* 20 291–302. 10.1016/j.devcel.2011.01.011 21397841

[B103] Li S. Wang R. Wang Y. Li H. Zheng J. Duan R. (2014). Receptors of the notch signaling pathway are associated with hemorrhage of brain arteriovenous malformations. *Mol. Med. Rep.* 9 2233–38. 10.3892/mmr.2014.2061 24643729

[B104] Lindahl P. Johansson B. R. Levéen P. Betsholtz C. (1997). Pericyte loss and microaneurysm formation in PDGF-B-deficient mice. *Science* 277 242–45. 10.1126/science.277.5323.242 9211853

[B105] Lindblom P. Gerhardt H. Liebner S. Abramsson A. Enge M. Hellstrom M. (2003). Endothelial PDGF-B retention is required for proper investment of pericytes in the microvessel wall. *Genes Dev.* 17 1835–40. 10.1101/gad.266803 12897053 PMC196228

[B106] López-Coviella I. Berse B. Krauss R. Thies R. S. Blusztajn J. K. (2000). Induction and maintenance of the neuronal cholinergic phenotype in the central nervous system by BMP-9. *Science* 289 313–16. 10.1126/science.289.5477.313 10894782

[B107] Lubeck B. A. Lapinski P. E. Bauler T. J. Oliver J. A. Hughes E. D. Saunders T. L. (2014). Blood vascular abnormalities in Rasa1R780Q knockin mice: Implications for the pathogenesis of capillary malformation–arteriovenous malformation. *Am. J. Pathol.* 184 3163–69. 10.1016/j.ajpath.2014.08.018 25283357 PMC4258499

[B108] Mahmoud M. Allinson K. R. Zhai Z. Oakenfull R. Ghandi P. Adams R. H. (2010). Pathogenesis of arteriovenous malformations in the absence of endoglin. *Circ. Res.* 106 1425–33. 10.1161/CIRCRESAHA.109.211037 20224041

[B109] Matsubara S. Mandzia J. L. ter Brugge K. Willinsky R. A. Faughnan M. E. (2000). Angiographic and clinical characteristics of patients with cerebral arteriovenous malformations associated with hereditary hemorrhagic telangiectasia. *AJNR* 21 1016–20. 10871005 PMC7973909

[B110] McAllister K. A. Grogg K. M. Johnson D. W. Gallione C. J. Baldwin M. A. Jackson C. E. (1994). Endoglin, a TGF-β binding protein of endothelial cells, is the gene for hereditary haemorrhagic telangiectasia type 1. *Nat. Genet.* 8 345–51. 10.1038/ng1294-345 7894484

[B111] Miller D. W. Graulich W. Karges B. Stahl S. Ernst M. Ramaswamy A. (1999). Elevated expression of endoglin, a component of the TGF-β-receptor complex, correlates with proliferation of tumor endothelial cells. *Int. J. Cancer* 81 568–72. 10225446 10.1002/(sici)1097-0215(19990517)81:4<568::aid-ijc11>3.0.co;2-x

[B112] Mohr J. P. Kejda-Scharler J. Spellman J. P. - (2013). Diagnosis and treatment of arteriovenous malformations. *Curr. Neurol. Neurosci. Rep.* 13:324. 10.1007/s11910-012-0324-1 23307509

[B113] Moya I. M. Umans L. Maas E. Pereira P. N. G. Beets K. Francis A. (2012). Stalk cell phenotype depends on integration of notch and Smad1/5 signaling cascades. *Dev. Cell* 22 501–14. 10.1016/j.devcel.2012.01.007 22364862 PMC4544746

[B114] Murphy P. A. Kim T. N. Huang L. Nielsen C. M. Lawton M. T. Adams R. H. (2014). Constitutively active Notch4 receptor elicits brain arteriovenous malformations through enlargement of capillary-like vessels. *Proc. Natl. Acad. Sci. U.S.A.* 111 18007–12. 10.1073/pnas.1415316111 25468970 PMC4273347

[B115] Murphy P. A. Lam M. T. Y. Wu X. Kim T. N. Vartanian S. M. Bollen A. W. (2008). Endothelial Notch4 signaling induces hallmarks of brain arteriovenous malformations in mice. *Proc. Natl. Acad. Sci. U.S.A.* 105 10901–6. 10.1073/pnas.0802743105 18667694 PMC2504798

[B116] Nadeem T. Bogue W. Bigit B. Cuervo H. (2020). Deficiency of notch signaling in pericytes results in arteriovenous malformations. *JCI Insight* 5:e125940. 10.1172/jci.insight.125940 33148887 PMC7710269

[B117] Nakao A. Imamura T. Souchelnytskyi S. Kawabata M. Ishisaki A. Oeda E. (1997). TGF-beta receptor-mediated signalling through Smad2, Smad3 and Smad4. *EMBO J.* 16 5353–62. 10.1093/emboj/16.17.5353 9311995 PMC1170167

[B118] Neuhaus H. Rosen V. Thies R. S. (1999). Heart specific expression of mouse BMP-10 a novel member of the TGF-beta superfamily. *Mech. Dev.* 80 181–84. 10.1016/s0925-4773(98)00221-4 10072785

[B119] Nielsen C. M. Cuervo H. Ding V. W. Kong Y. Huang E. J. Wang R. A. (2014). Deletion of Rbpj from postnatal endothelium leads to abnormal arteriovenous shunting in mice. *Development* 141 3782–92. 10.1242/dev.108951 25209249 PMC4197591

[B120] Noguera-Troise I. Daly C. Papadopoulos N. J. Coetzee S. Boland P. Gale N. W. (2006). Blockade of Dll4 inhibits tumour growth by promoting non-productive angiogenesis. *Nature* 444 1032–37. 10.1038/nature05355 17183313

[B121] Oh S. P. Seki T. Goss K. A. Imamura T. Yi Y. Donahoe P. K. (2000). Activin receptor-like kinase 1 modulates transforming growth factor-beta 1 signaling in the regulation of angiogenesis. *Proc. Natl. Acad. Sci. U.S.A.* 97 2626–31. 10.1073/pnas.97.6.2626 10716993 PMC15979

[B122] Ola R. Dubrac A. Han J. Zhang F. Fang J. S. Larrivée B. (2016). PI3 kinase inhibition improves vascular malformations in mouse models of hereditary haemorrhagic telangiectasia. *Nat. Commun.* 7:13650. 10.1038/ncomms13650 27897192 PMC5141347

[B123] Ola R. Künzel S. H. Zhang F. Genet G. Chakraborty R. Pibouin-Fragner L. (2018). SMAD4 prevents flow induced arteriovenous malformations by inhibiting casein kinase 2. *Circulation* 138 2379–94. 10.1161/CIRCULATIONAHA.118.033842 29976569 PMC6309254

[B124] Oosting S. Nagengast W. de Vries E. (2009). More on bevacizumab in hereditary hemorrhagic telangiectasia. *N. Engl. J. Med.* 361:931. 10.1056/NEJMc091271 19710496

[B125] Ouarné M. Pena A. Franco C. A. (2021). From remodeling to quiescence: The transformation of the vascular network. *Cells Dev. Quan. Cell Dev. Biol.* 168:203735. 10.1016/j.cdev.2021.203735 34425253

[B126] Paredes I. Himmels P. Ruiz de Almodóvar C. (2018). Neurovascular communication during CNS development. *Dev. Cell* 45 10–32. 10.1016/j.devcel.2018.01.023 29634931

[B127] Park E. S. Kim S. Huang S. Yoo J. Y. Körbelin J. Lee T. J. (2021). Selective endothelial hyperactivation of oncogenic KRAS induces brain arteriovenous malformations in mice. *Ann. Neurol.* 89 926–41. 10.1002/ana.26059 33675084

[B128] Park H. Furtado J. Poulet M. Chung M. Yun S. Lee S. (2021). Defective flow-migration coupling causes arteriovenous malformations in hereditary hemorrhagic telangiectasia. *Circulation* 144 805–22. 10.1161/CIRCULATIONAHA.120.053047 34182767 PMC8429266

[B129] Park S. DiMaio T. A. Liu W. Wang S. Sorenson C. M. Sheibani N. (2013). Endoglin regulates the activation and quiescence of endothelium by participating in canonical and non-canonical TGF-β signaling pathways. *J. Cell Sci.* 126 1392–1405. 10.1242/jcs.117275 23418351 PMC3644140

[B130] Park S. Ok M. Wankhede Y. J. Lee E.-J. Choi N. Fliess S.-W. (2009). Real-time imaging of de novo arteriovenous malformation in a mouse model of hereditary hemorrhagic telangiectasia. *J. Clin. Investig.* 119 3487–96. 10.1172/JCI39482 19805914 PMC2769195

[B131] Park S. O. Lee Y. J. Seki T. Hong K. H. Fliess N. Jiang Z. (2008). ALK5- and TGFBR2-independent role of ALK1 in the pathogenesis of hereditary hemorrhagic telangiectasia type 2. *Blood* 111 633–42. 10.1182/blood-2007-08-107359 17911384 PMC2200847

[B132] Parmar K. M. Larman H. B. Dai G. Zhang Y. Wang E. T. Moorthy S. N. (2006). Integration of flow-dependent endothelial phenotypes by kruppel-like factor 2. *J. Clin. Investig.* 116 49–58. 10.1172/JCI24787 16341264 PMC1307560

[B133] Peacock H. M. Tabibian A. Criem N. Caolo V. Hamard L. Deryckere A. (2020). Impaired SMAD1/5 mechanotransduction and Cx37 (Connexin37) expression enable pathological vessel enlargement and shunting. *Arterioscler. Thromb. Vasc. Biol.* 40:e87–104. 10.1161/ATVBAHA.119.313122 32078368

[B134] Peguera B. Segarra M. Acker-Palmer A. (2021). Neurovascular crosstalk coordinates the central nervous system development. *Curr. Opin. Neurobiol. Mol. Neurosci.* 69 202–13. 10.1016/j.conb.2021.04.005 34077852 PMC8411665

[B135] Pitulescu M. E. Schmidt I. Giaimo B. D. Antoine T. Berkenfeld F. Ferrante F. (2017). Dll4 and notch signalling couples sprouting angiogenesis and artery formation. *Nat. Cell Biol.* 19 915–27. 10.1038/ncb3555 28714968

[B136] Prigoda N. L. Savas S. Abdalla S. A. Piovesan B. Rushlow D. Vandezande K. (2006). Hereditary haemorrhagic telangiectasia: Mutation detection, test sensitivity and novel mutations. *J. Med. Genet.* 43 722–28. 10.1136/jmg.2006.042606 16690726 PMC2564570

[B137] Revencu N. Boon L. M. Mulliken J. B. Enjolras O. Cordisco M. R. Burrows P. E. (2008). Parkes weber syndrome, vein of galen aneurysmal malformation, and other fast-flow vascular anomalies are caused by RASA1 mutations. *Hum. Mutat.* 29 959–65. 10.1002/humu.20746 18446851

[B138] Ricard N. Ciais D. Levet S. Subileau M. Mallet C. Zimmers T. A. (2012). BMP9 and BMP10 are critical for postnatal retinal vascular remodeling. *Blood* 119 6162–71. 10.1182/blood-2012-01-407593 22566602 PMC3383024

[B139] Robert F. D-Castan A. Bailly S. Girod S. D. Feige J.-J. (2020). Future treatments for hereditary hemorrhagic telangiectasia. *Orphanet J. Rare Dis.* 15:4. 10.1186/s13023-019-1281-4 31910860 PMC6945546

[B140] Rochon E. R. Menon P. G. Roman B. L. (2016). Alk1 controls arterial endothelial cell migration in lumenized vessels. *Development* 143 2593–2602. 10.1242/dev.135392 27287800 PMC4958337

[B141] Rochon E. R. Wright D. S. Schubert M. M. Roman B. L. (2015). Context-specific interactions between notch and ALK1 cannot explain ALK1-associated arteriovenous malformations. *Cardiovasc. Res.* 107 143–52. 10.1093/cvr/cvv148 25969392 PMC4498135

[B142] Rohn B. Haenggi D. Etminan N. Kunz M. Turowski B. Steiger H.-J. (2014). Epilepsy, headache, and quality of life after resection of cerebral arteriovenous malformations. *J. Neurol. Surg. Part A* 75 282–88. 10.1055/s-0033-1358611 24623000

[B143] Roman B. L. Hinck A. P. (2017). ALK1 signaling in development and disease: New paradigms. *Cell. Mol. Life Sci.* 74 4539–60. 10.1007/s00018-017-2636-4 28871312 PMC5687069

[B144] Roman B. L. Pham V. N. Lawson N. D. Kulik M. Childs S. Lekven A. C. (2002). Disruption of acvrl1 increases endothelial cell number in zebrafish cranial vessels. *Development* 129 3009–19. 10.1242/dev.129.12.3009 12050147

[B145] Ruiz S. Zhao H. Chandakkar P. Chatterjee P. K. Papoin J. Blanc L. (2016). A mouse model of hereditary hemorrhagic telangiectasia generated by transmammary-delivered immunoblocking of BMP9 and BMP10. *Sci. Rep.* 5:37366. 10.1038/srep37366 27874028 PMC5118799

[B146] Ruiz S. Zhao H. Chandakkar P. Papoin J. Choi H. Kitabayashi A. N. (2020). Correcting Smad1/5/8, MTOR, and VEGFR2 treats pathology in hereditary hemorrhagic telangiectasia models. *J. Clin. Investig.* 130 942–57. 10.1172/JCI127425 31689244 PMC6994128

[B147] Saleh M. Carter M. T. Latino G. A. Dirks P. Ratjen F. (2013). Brain arteriovenous malformations in patients with hereditary hemorrhagic telangiectasia: Clinical presentation and anatomical distribution. *Pediatr. Neurol.* 49 445–50. 10.1016/j.pediatrneurol.2013.07.021 24080277

[B148] Schuermann A. Helker C. S. M. Herzog W. (2014). Angiogenesis in zebrafish. *Semin. Cell Dev. Biol.* 31 106–14. 10.1016/j.semcdb.2014.04.037 24813365

[B149] Seki T. Yun J. Oh S. P. (2003). Arterial endothelium-specific activin receptor-like kinase 1 expression suggests its role in arterialization and vascular remodeling. *Circ. Res.* 93 682–89. 10.1161/01.RES.0000095246.40391.3B 12970115

[B150] Serini G. Napione L. Arese M. Bussolino F. (2008). Besides adhesion: New perspectives of integrin functions in angiogenesis. *Cardiovasc. Res.* 78 213–22. 10.1093/cvr/cvn045 18285512

[B151] Serra H. Chivite I. A-Urarte A. Soler A. Sutherland J. D. A-Aristorena A. (2015). PTEN mediates notch-dependent stalk cell arrest in angiogenesis. *Nat. Commun.* 6:7935. 10.1038/ncomms8935 26228240 PMC5426521

[B152] Shaligram S. S. Winkler E. Cooke D. Su H. (2019). Risk factors for hemorrhage of brain arteriovenous malformation. *CNS Neurosci. Ther.* 25 1085–95. 10.1111/cns.13200 31359618 PMC6776739

[B153] Shovlin C. L. (2010). Hereditary haemorrhagic telangiectasia: Pathophysiology, diagnosis and treatment. *Blood Rev.* 24 203–19. 10.1016/j.blre.2010.07.001 20870325

[B154] Siegenthaler J. A. Sohet F. Daneman R. (2013). ‘Sealing off the CNS’: Cellular and molecular regulation of blood–brain barriergenesis. *Curr. Opin. Neurobiol.* 23 1057–64. 10.1016/j.conb.2013.06.006 23867075 PMC4061913

[B155] Simons M. Gordon E. Welsh L. C. (2016). Mechanisms and regulation of endothelial VEGF receptor signalling. *Nat. Rev. Mol. Cell Biol.* 17 611–25. 10.1038/nrm.2016.87 27461391

[B156] Singh E. Redgrave R. E. Phillips H. M. Arthur H. M. (2020). Arterial endoglin does not protect against arteriovenous malformations. *Angiogenesis* 23 559–66. 10.1007/s10456-020-09731-z 32506200 PMC7524831

[B157] Skaro A. I. Marotta P. J. McAlister V. C. (2006). Regression of cutaneous and gastrointestinal telangiectasia with sirolimus and aspirin in a patient with hereditary hemorrhagic telangiectasia. *Ann. Intern. Med.* 144 226–27. 10.7326/0003-4819-144-3-200602070-00030 16461979

[B158] Snodgrass R. O. Chico T. J. A. Arthur H. M. (2021). Hereditary haemorrhagic telangiectasia, an inherited vascular disorder in need of improved evidence-based pharmaceutical interventions. *Genes* 12:174. 33513792 10.3390/genes12020174 PMC7911152

[B159] Somi S. Buffing A. A. M. Moorman A. F. M. Van Den Hoff M. J. B. (2004). Expression of bone morphogenetic protein-10 MRNA during chicken heart development. *Anat. Rec. Part A Discov. Mol. Cell. Evol. Biol.* 279 579–82. 10.1002/ar.a.20052 15224399

[B160] Srinivasan S. Hanes M. A. Dickens T. Porteous M. E. M. Oh S. P. Hale L. P. (2003). A mouse model for hereditary hemorrhagic telangiectasia (HHT) type 2. *Hum. Mol. Genet.* 12 473–82. 10.1093/hmg/ddg050 12588795

[B161] Suchting S. Freitas C. Noble F. L. Benedito R. Bréant C. Duarte A. (2007). The notch ligand delta-like 4 negatively regulates endothelial tip cell formation and vessel branching. *Proc. Natl. Acad. Sci. U.S.A.* 104 3225–30. 10.1073/pnas.0611177104 17296941 PMC1805603

[B162] Sugden W. W. Meissner R. Wilmsen T. A. Tsaryk R. Leonard E. V. Bussmann J. (2017). Endoglin controls blood vessel diameter through endothelial cell shape changes in response to haemodynamic cues. *Nat. Cell Biol.* 19 653–65. 10.1038/ncb3528 28530658 PMC5455977

[B163] Swiatek P. J. Lindsell C. E. del Amo F. F. Weinmaster G. Gridley T. (1994). Notch1 Is essential for postimplantation development in mice. *Genes Dev.* 8 707–19. 10.1101/gad.8.6.707 7926761

[B164] Tan W. H. Popel A. S. Gabhann F. M. (2013). Computational model of VEGFR2 pathway to ERK activation and modulation through receptor trafficking. *Cell. Signal.* 25 2496–510. 10.1016/j.cellsig.2013.08.015 23993967 PMC3865527

[B165] Tata M. Ruhrberg C. Fantin A. (2015). Vascularisation of the central nervous system. *Mech. Dev.* 138 26–36. 10.1016/j.mod.2015.07.001 26222953 PMC4678116

[B166] Thalgott J. H. D-Santos-Luis D. Hosman A. E. Martin S. Lamandé N. Bracquart D. (2018). Decreased expression of vascular endothelial growth factor receptor 1 contributes to the pathogenesis of hereditary hemorrhagic telangiectasia type 2. *Circulation* 138 2698–2712. 10.1161/CIRCULATIONAHA.117.033062 30571259

[B167] Torsney E. Charlton R. Diamond A. G. Burn J. Soames J. V. Arthur H. M. (2003). Mouse model for hereditary hemorrhagic telangiectasia has a generalized vascular abnormality. *Circulation* 107 1653–57. 10.1161/01.CIR.0000058170.92267.00 12668501

[B168] Torsney E. Parums R. C. D. Collis M. Arthur H. M. (2002). Inducible expression of human endoglin during inflammation and wound healing *in vivo*. *Inflamm. Res.* 51 464–70. 10.1007/PL00012413 12365720

[B169] Townson S. A. M-Hackert E. Greppi C. Lowden P. Sako D. Liu J. (2012). Specificity and structure of a high affinity activin receptor-like kinase 1 (ALK1) signaling complex. *J. Biol. Chem.* 287 27313–25. 10.1074/jbc.M112.377960 22718755 PMC3431715

[B170] Trindade A. Kumar S. R. Scehnet J. S. Lopes-da-Costa L. Becker J. Jiang W. (2008). Overexpression of delta-like 4 induces arterialization and attenuates vessel formation in developing mouse embryos. *Blood* 112 1720–29. 10.1182/blood-2007-09-112748 18559979 PMC2518882

[B171] Tual-Chalot S. Mahmoud M. Allinson K. R. Redgrave R. E. Zhai Z. Oh S. P. (2014). Endothelial depletion of acvrl1 in mice leads to arteriovenous malformations associated with reduced endoglin expression. *PLoS One* 9:e98646. 10.1371/journal.pone.0098646. 24896812 PMC4045906

[B172] Tual-Chalot S. Oh P. Arthur H. (2015). Mouse models of hereditary haemorrhagic telangiectasia: Recent advances and future challenges. *Front. Genet.* 6:25. 10.3389/fgene.2015.00025 25741358 PMC4332371

[B173] Tzima E. Kiosses W. B. del Pozo M. A. Schwartz M. A. (2003). Localized Cdc42 activation, detected using a novel assay, mediates microtubule organizing center positioning in endothelial cells in response to fluid shear stress. *J. Biol. Chem.* 278 31020–3. 10.1074/jbc.M301179200 12754216

[B174] Urness L. D. Sorensen L. K. Li D. Y. (2000). Arteriovenous malformations in mice lacking activin receptor-like kinase-1. *Nat. Genet.* 26 328–31. 10.1038/81634 11062473

[B175] Valdimarsdottir G. M-J Goumans Rosendahl A. Brugman M. Itoh S. Lebrin F. (2002). Stimulation of Id1 expression by bone morphogenetic protein is sufficient and necessary for bone morphogenetic protein-induced activation of endothelial cells. *Circulation* 106 2263–70. 10.1161/01.cir.0000033830.36431.46 12390958

[B176] Van Gieson E. J. Murfee W. L. Skalak T. C. Price R. J. (2003). Enhanced smooth muscle cell coverage of microvessels exposed to increased hemodynamic stresses *in vivo*. *Circ. Res.* 92 929–36. 10.1161/01.RES.0000068377.01063.79 12663481

[B177] Walcott B. P. Winkler E. A. Zhou S. Birk H. Guo D. Koch M. J. (2018). Identification of a rare BMP pathway mutation in a non-syndromic human brain arteriovenous malformation via exome sequencing. *Hum. Genome Var.* 5:18001. 10.1038/hgv.2018.1 29844917 PMC5966745

[B178] Walker E. J. Su H. Shen F. Choi E.-J. Oh S. P. Chen G. (2011). Arteriovenous malformation in the adult mouse brain resembling the human disease. *Ann. Neurol.* 69 954–62. 10.1002/ana.22348 21437931 PMC3117949

[B179] Willemse R. B. Mager J. J. Westermann C. J. Overtoom T. T. Mauser H. Wolbers J. G. (2000). Bleeding risk of cerebrovascular malformations in hereditary hemorrhagic telangiectasia. *J. Neurosurg.* 92 779–84. 10.3171/jns.2000.92.5.0779 10794291

[B180] Winkler E. A. Birk H. Burkhardt J.-K. Chen X. Yue J. K. Guo D. (2018). Reductions in brain pericytes are associated with arteriovenous malformation vascular instability. *J. Neurosurg.* 129 1464–74. 29303444 10.3171/2017.6.JNS17860 PMC6033689

[B181] Wooderchak-Donahue W. L. McDonald J. O’Fallon B. Upton P. D. Li W. Roman B. L. (2013). BMP9 mutations cause a vascular-anomaly syndrome with phenotypic overlap with hereditary hemorrhagic telangiectasia. *Am. J. Hum. Genet.* 93 530–37. 10.1016/j.ajhg.2013.07.004 23972370 PMC3769931

[B182] Xu C. Hasan S. S. Schmidt I. Rocha S. F. Pitulescu M. E. Bussmann J. (2014). Arteries are formed by vein-derived endothelial tip cells. *Nat. Commun.* 5:5758. 10.1038/ncomms6758 25502622 PMC4275597

[B183] Xue Y. Gao X. Lindsell C. E. Norton C. R. Chang B. Hicks C. (1999). Embryonic lethality and vascular defects in mice lacking the notch ligand jagged1. *Hum. Mol. Genet.* 8 723–30. 10.1093/hmg/8.5.723 10196361

[B184] Yamada S. Takagi Y. Nozaki K. Kikuta K.-I. Hashimoto N. (2007). Risk factors for subsequent hemorrhage in patients with cerebral arteriovenous malformations. *J. Neurosurg.* 107 965–72. 10.3171/JNS-07/11/0965 17977268

[B185] Yao Y. Jumabay M. Wang A. Boström K. I. (2011). Matrix Gla protein deficiency causes arteriovenous malformations in mice. *J. Clin. Investig.* 121 2993–3004. 10.1172/JCI57567 21765215 PMC3148746

[B186] Yao Y. Yao J. Radparvar M. Blazquez-Medela A. M. Guihard P. J. Jumabay M. (2013). Reducing Jagged 1 and 2 levels prevents cerebral arteriovenous malformations in matrix Gla protein deficiency. *Proc. Natl. Acad. Sci. U.S.A.* 110 19071–76. 10.1073/pnas.1310905110 24191040 PMC3839731

[B187] Zhu W. Chen W. Zou D. Wang L. Bao C. Zhan L. (2018). Thalidomide reduces hemorrhage of brain arteriovenous malformations in a mouse model. *Stroke* 49 1232–40. 10.1161/STROKEAHA.117.020356 29593101 PMC5916043

[B188] ZhuGe Q. Wu Z. Huang L. Zhao B. Zhong M. Zheng W. (2013). Notch4 is activated in endothelial and smooth muscle cells in human brain arteriovenous malformations. *J. Cell. Mol. Med.* 17 1458–64. 10.1111/jcmm.12115 24373503 PMC3877925

[B189] ZhuGe Q. Zhong M. Zheng W. Yang G.-Y. Mao X. Xie L. (2009). Notch-1 signalling is activated in brain arteriovenous malformations in humans. *Brain* 132 3231–41. 10.1093/brain/awp246 19812212 PMC2792368

